# Structural Pharmacology of TRPV4 Antagonists

**DOI:** 10.1002/advs.202401583

**Published:** 2024-04-24

**Authors:** Junping Fan, Chang Guo, Daohong Liao, Han Ke, Jing Lei, Wenjun Xie, Yuliang Tang, Makoto Tominaga, Zhuo Huang, Xiaoguang Lei

**Affiliations:** ^1^ Beijing National Laboratory for Molecular Sciences Key Laboratory of Bioorganic Chemistry and Molecular Engineering of Ministry of Education College of Chemistry and Molecular Engineering Peking‐Tsinghua Center for Life Sciences Peking University Beijing 100871 China; ^2^ State Key Laboratory of Natural and Biomimetic Drugs Department of Molecular and Cellular Pharmacology School of Pharmaceutical Sciences Peking University Health Science Center Beijing 100191 China; ^3^ Iongen Therapeutics Co. Ltd. Nanjing 211151 China; ^4^ Division of Cell Signaling National Institute for Physiological Sciences Thermal Biology Group Exploratory Research Center on Life and Living Systems National Institutes of Natural Sciences Okazaki 444‐8787 Japan; ^5^ Nagoya Advanced Research and Developmet Center Nagoya City University Nagoya 467‐8601 Japan

**Keywords:** antagonist, cryo‐EM structure, drug discovery, structural pharmacology, TRPV4

## Abstract

The nonselective calcium‐permeable Transient Receptor Potential Cation Channel Subfamily V Member4 (TRPV4) channel regulates various physiological activities. Dysfunction of TRPV4 is linked to many severe diseases, including edema, pain, gastrointestinal disorders, lung diseases, and inherited neurodegeneration. Emerging TRPV4 antagonists show potential clinical benefits. However, the molecular mechanisms of TRPV4 antagonism remain poorly understood. Here, cryo‐electron microscopy (cryo‐EM) structures of human TRPV4 are presented in‐complex with two potent antagonists, revealing the detailed binding pockets and regulatory mechanisms of TRPV4 gating. Both antagonists bind to the voltage‐sensing‐like domain (VSLD) and stabilize the channel in closed states. These two antagonists induce TRPV4 to undergo an apparent fourfold to twofold symmetry transition. Moreover, it is demonstrated that one of the antagonists binds to the VSLD extended pocket, which differs from the canonical VSLD pocket. Complemented with functional and molecular dynamics simulation results, this study provides crucial mechanistic insights into TRPV4 regulation by small‐molecule antagonists, which may facilitate future drug discovery targeting TRPV4.

## Graphical abstract

The cryo‐EM structures of the human TRPV4 ion channel in complex with two potent small‐molecule antagonists provide mechanistic insights into the molecular basis of TRPV4 inhibition. The structural and functional elucidation of the antagonists mechanisms of action presented in this study will significantly facilitate the drug discovery targeting the TRPV4 channel.

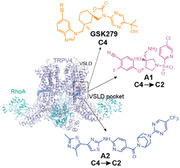

## 1 Introduction

Transient Receptor Potential Cation Channel Subfamily V Member 4 (TRPV4) is a ubiquitously expressed, nonselective, Ca^2+^‐permeable cation channel^[^ ^1^ ^]^ that belongs to the TRP family. TRPV4 is widely expressed across different tissues, such as the skin, vascular tissue, nervous system, lungs, kidneys, and bones, and contributes to diverse functions.^[^ ^2^ ^]^ This polymodal protein can be activated not only by environmental stimuli, including temperature, hypoosmotic conditions, and mechanical stress,^[^ ^3^ ^]^ but also by endogenous agonists such as anandamide and arachidonic acid.^[^ ^4^ ^]^ TRPV4 is involved in various physiological processes, such as osmoregulation, inflammation, nociception, and temperature sensation,^[^ ^1^ ^]^ and plays a vital role in human health. Dysfunction of the TRPV4 channel is implicated in various diseases,^[^ ^5^ ^]^ including skeletal dysplasias; neuromuscular disorders, such as Charcot‐Marie‐Tooth disease; cardiovascular diseases like hypertension; and respiratory disorders, including pulmonary edema, osteoarthritis, and renal disorders.^[^ ^6^ ^]^ The diverse involvement of TRPV4 underscores its significance in potential therapeutic strategies for these conditions.^[^ ^3^ ^]^ Because of the critical function of TRPV4 and its close association with diseases, TRPV4 represents a valuable therapeutic target.^[^ ^7^ ^]^ Several lead compounds targeting TRPV4 have shown promising therapeutic effects.^[^ ^8^ ^]^ One of the compounds, GSK2798745 (GSK279),^[^ ^9^ ^]^ developed by GSK, is undergoing clinical trials to treat pulmonary edema associated with congestive heart failure. It is a first‐in‐class, orally active, highly potent, and selective blocker of the TRPV4 channel.^[^ ^10^ ^]^ This makes GSK279 the first TRPV4 antagonist to be studied in humans.^[^ ^8^ ^]^ Another preclinical candidate TRPV4 inhibitor, GSK3527497 (antagonist 1, A1),^[^ ^11^ ^]^ is a pyrrolidine diol derivative and backup molecule for the clinical lead compound GSK279. In addition, Shionogi & Co developed the compound 2′,4′‐dimethyl‐[4,5′‐bithiazol]−2‐yl amino derivative^[^ ^12^ ^]^ (antagonist 2, A2), which is an orally bioavailable TRPV4 antagonist for pain treatment. Both small molecules have shown high potency in antagonizing TRPV4. However, the precise binding modes of the two TRPV4 antagonists and their inhibitory mechanisms remain unclear.

Cryo‐electron microscopy (cryo‐EM) analysis has been a powerful tool for studying channel regulation by small molecules. For TRPV channels, many compound binding modes for TRPV1‐3 have been investigated. However, only a few antagonists and agonists of TRPV4 were reported recently.^[^ ^13^ ^]^ In addition, several structures of TRPV2^[^ ^14^ ^]^ and TRPV3^[^ ^15^ ^]^ in twofold (C2) rather than fourfold (C4) symmetry have recently been reported; for TRPV4, only subtle changes were reported.^[^ ^13^ ^b]^ In this study, we determined two cryo‐EM structures of TRPV4 in complexes with two antagonists, respectively. Our structural, functional, and computational results elucidated the binding modes of the antagonists and their antagonistic effect on TRPV4, providing valuable insights into TRPV4 inhibition. Furthermore, substantial C4‐ to C2‐ symmetry reductions and conformational changes were observed in the A1 and A2 antagonist‐bound structures, suggesting that the symmetry alteration also exists in TRPV4 channel regulation.

## 2 Results and Discussion

### 2.1 Two Compounds Inhibit TRPV4

**Figure 1:**
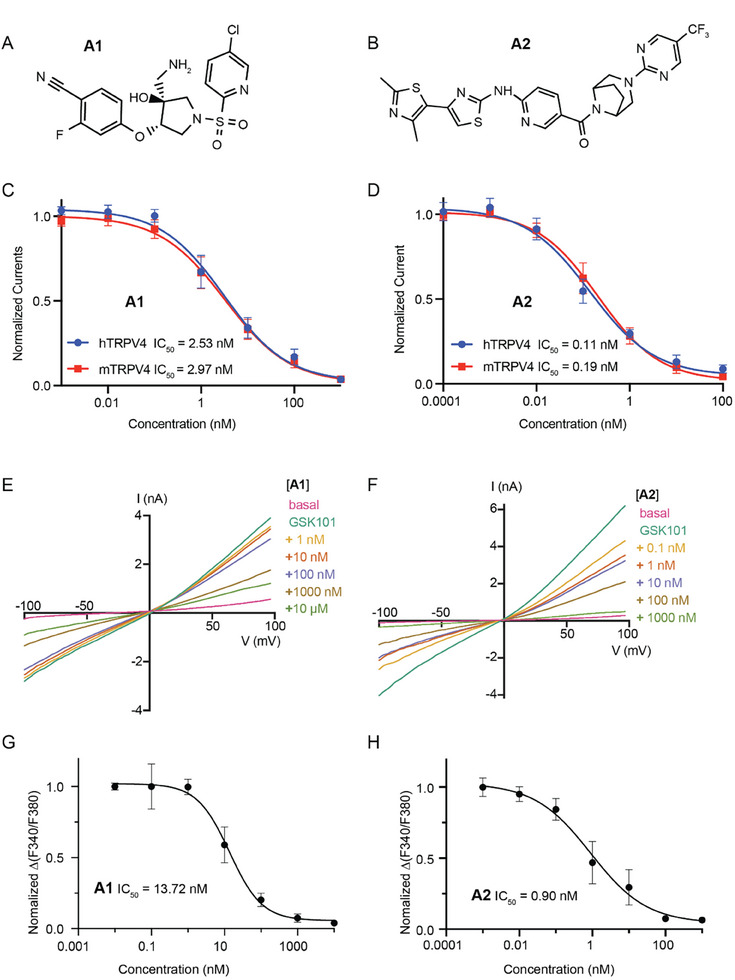
A1 and A2 are two potent antagonists of TRPV4. A,B) The chemical structures of the TRPV4 antagonists A1 and A2. C,D) Curve fitting of dose‐dependent inhibition of 10 nm GSK101‐evoked wild‐type TRPV4 current by A1 (C hTRPV4 IC_50_ = 2.53 ± 0.52 nm, *n* = 4; mTRPV4 IC_50_ = 2.97 ± 0.58 nm, *n* = 4) or A2 (D hTRPV4 IC_50_ = 0.11 ± 0.02 nm, *n* = 4; mTRPV4 IC_50_ = 0.19 ± 0.03 nm, *n* = 4). E,F) *I*–*V* curves of currents of keratinocytes inhibited by A1 (E) or A2 (F) (+, with 300 nmm GSK101). G,H) Dose‐response curves of endogenous mTRPV4 against A1 (G) or A2 (H) recorded by Ca^2+^ imaging of keratinocytes. Error bars, s.e.m.

We tested the inhibitory effects of A1 and A2 on human TRPV4 (hTRPV4) using the whole‐cell patch‐clamp recording technique. GSK1016790A (GSK101), a commonly used TRPV4 activator,^[^ ^16^ ^]^ was used to activate hTRPV4 transiently expressed in Chinese hamster ovary (CHO) cells (Figure S1A‐C, Supporting Information), yielding a half maximal effective concentration (EC_50_) of 1.78–2.14 nm at ±60 mv (Figure S1D, Supporting Information). The concentration of GSK101 was set at 10 nm to elicit ≈80% of the peak current, which is sufficient to evaluate the inhibitory effects of the two antagonists. The potent inhibitory effects of the antagonists on hTPRV4 were consistent when the membrane potential was held at ±60 mV (Figure S1E‐H, Supporting Information); therefore, in the following experiments, +60 mV was used. We found that both compounds gradually inhibited hTRPV4 in a dose‐dependent manner, resulting in half‐maximal inhibitory concentration (IC_50_) values of 2.53 and 0.11 nm for compounds A1 and A2, respectively (**Figure** **1**A‐D). In line with the findings of previous studies,^[^ ^9^, ^11^, ^12^ ^]^ these results confirmed that compounds A1 and A2 are indeed potent inhibitors of hTRPV4. We further evaluated their inhibitory effects on mouse TRPV4 (mTRPV4) because most animal experiments were conducted using mice. The result showed that A1 and A2 had a similar impact on mTRPV4 (Figure 1C,D; Figure S1E‐H, Supporting Information). Subsequently, we tested the inhibitory effects of A1 and A2 on primary skin keratinocyte cells, which have been used to test endogenous TRPV4.^[^ ^17^ ^]^ When exposed to 300 nm GSK101, keratinocyte cells exhibited robust currents. In line with the inhibitory effects of A1 or A2 on hTRPV4 overexpressed in CHO cells, the co‐application of gradient A1 or A2 gradually reduced the currents of endogenous TRPV4 elicited by GSK101 (Figure 1E,F; Figure S1I‐K, Supporting Information). We also evaluated the inhibition of endogenous mTRPV4 by calcium imaging (Figure S1L‐N, Supporting Information). The F340/F380 ratio changes were normalized to the values without A1 or A2 administration. The obtained IC_50_ values were 13.72 nm for A1 and 0.9 nm for A2 (Figure 1G,H), comparable with the values obtained from CHO cells. The above results indicate that A1 and A2 are potent antagonists of TRPV4.

### 2.2 Cryo‐EM Structures of hTRPV4 in Complex with Two Different Antagonists

**Figure 2:**
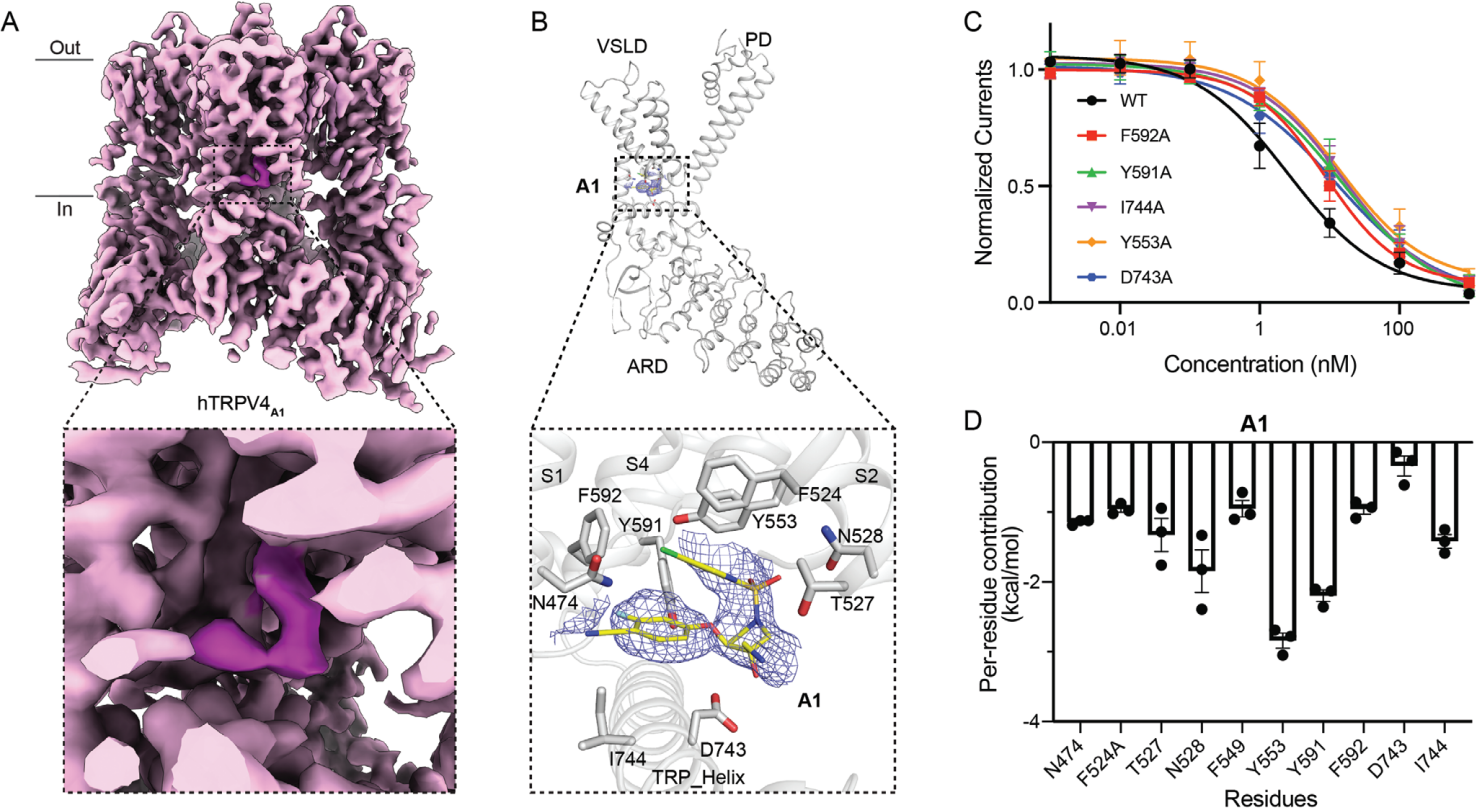
Detailed binding sites for A1 in hTRPV4. A) Cryo‐EM reconstruction of hTRPV4 in the ligand A1‐bound form. The black dashed square indicates the region shown in the enlarged view to provide the detailed binding pocket for the antagonists. The electron density of A1 inside the VSLD pocket is highlighted for clarity. B) A single chain of hTRPV4_A1_ viewed parallel to the membrane. The close‐up view of the detailed interactions between hTRPV4 and A1 is at the bottom. A1 is shown as sticks and is colored with carbon atoms in yellow, oxygen in red, and nitrogen in blue. The density around the antagonist was contoured at 5σ (blue mesh). The transmembrane segments S1‐S4 and the TRP helix are colored in gray. The side chains of key residues interacting with A1 are shown as sticks. C) Curve fitting of the dose‐dependent inhibition of 10 nm GSK101‐evoked current by A1 on various hTRPV4 mutants compared with hTRPV4^WT^. (Y553A, IC_50_ = 13.94 ± 4.10 nm, *n* = 4; Y591A, IC_50_ = 15.33 ± 4.12 nm, *n* = 4; F592A, IC_50_ = 8.81 ± 1.09 nm, *n* = 4; D743A, IC_50_ = 12.08 ± 3.08 nm, *n* = 4; I744A, IC_50_ = 15.60 ± 2.95 nm, *n* = 4). D) Per‐residues binding energy for A1 calculated by MM‐GBSA, Error bars, s.e.m.

**Figure 3:**
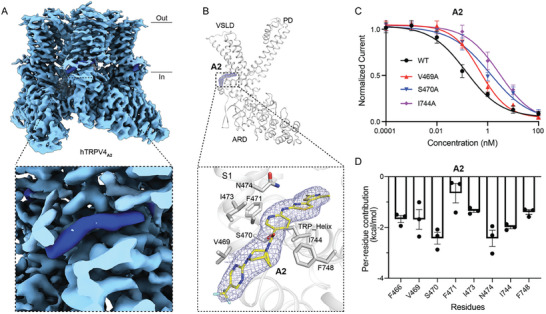
Detailed binding sites for A2 in hTRPV4. A) Cryo‐EM reconstruction of hTRPV4 in the ligand A2 bound form. The black dashed square indicates the region shown in the enlarged view to provide the detailed binding pocket for the antagonists. The electron density of A2 inside the VSLD pocket is highlighted for clarity. B) A single chain of hTRPV4_A2_ viewed parallel to the membrane. At the bottom are close‐up views of the detailed interactions between hTRPV4 and A2. A2 is shown as sticks and is colored with carbon atoms in yellow, oxygen in red, and nitrogen in blue. The density around A2 was contoured at 5σ (blue mesh). S1‐S4 and the TRP helix are colored in grey. The side chains of key residues interacting with A2 are shown in the sticks. C) Curve fitting of the dose‐dependent inhibition of 10 nm GSK101‐evoked current by A2 on various hTRPV4 mutants compared with hTRPV4^WT^. (V469A, IC_50_ = 0.42 ± 0.06 nm, *n* = 4; S470A, IC_50_ = 1.00 ± 0.30 nm, *n* = 4; I744A, IC_50_ = 3.00 ± 1.01 nm, *n* = 4). D) Per‐residues binding energy for A2 calculated by MM‐GBSA, Error bars, s.e.m.

To unveil the molecular mechanisms of these two compounds in inhibiting hTRPV4, we sought to determine the hTRPV4‐antagonist complex structures by cryo‐EM single‐particle analysis. Full‐length hTRPV4 protein samples were purified in the presence of each of the two antagonists at 10 µm individually (Figure S2A,B, Supporting Information), and the purified samples were further incubated with 100 µm antagonists to saturate the binding sites. We next collected cryo‐EM data from the purified hTRPV4 samples. We obtained final EM maps of hTRPV4 in complex with compounds A1 (hTRPV4_A1_) and A2 (hTRPV4_A2_) at resolutions of 3.74 Å (**Figure** **2**A; Figure S3A‐D, Supporting Information) and 3.21 Å (**Figure** **3**A; Figure S3E‐K, Supporting Information), respectively. For comparison, we also determined the cryo‐EM structure of hTRPV4 with GSK279 (hTRPV4_GSK279_) with a resolution of 3.45 Å (Figure S4A‐D, Supporting Information). These high‐quality EM maps allowed the reliable model building of hTRPV4. Notably, unambiguous EM densities of different shapes were observed in the voltage sensing‐like domains (VSLDs) of the hTRPV4 maps (Figures 2A and 3A; Figure S4E, Supporting Information), which are distinct from the possible lipid or Ca^2+^ densities found in the previously reported TRP structures.^[^ ^18^ ^]^ Importantly, the EM densities can reasonably accommodate the corresponding antagonists, indicating that all three antagonists bind in partially overlapping but not identical pockets inside the VSLD (Figures 2A and 3A; Figure S4E, Supporting Information). Like other TRPV channels, the triangle‐shaped VSLD pocket of TRPV4 comprises S1‐S4 bundles and the TRP helix.^[^ ^18a^ ^]^ Among the three structures, the reversed C‐shaped compounds GSK279 and A1 are almost buried in the VSLD pocket (Figure 2A,B; Figure S4E,F, Supporting Information). In contrast, the surfboard‐shaped compound A2 sticks half of its body into the VSLD pocket, with the remaining parts sitting outside (Figure 3A,B).

The overall structures of hTRPV4_GSK279_, hTRPV4_A1_, and hTRPV4_A2_ closely resemble the closed‐state hTRPV3^[^ ^15^ ^]^ (PDB code: 7XJ0) with Cα backbone root mean squared deviation (RMSD) values of 2.5, 3.8, and 2.8 Å, respectively (Figure S5, Supporting Information), suggesting that these three inhibitor‐bound hTRPV4 structures were captured in the closed‐state. In preparing this manuscript, Kwon et al.^[^ ^13^ ^a]^ also reported the complex structure of hTRPV4 with GSK279, which is very similar to our hTRPV4_GSK279_ structure with an RMSD of 0.86 Å. However, the hTRPV4_A1_ and hTRPV4_A2_ structures adopt a C2‐symmetric configuration rather than C4 in the hTRPV4_GSK279_ structures. A similar C4‐ to C2‐ symmetry transition was observed in our previous hTRPV3‐G537S structure upon binding the inhibitor Trpvicin.^[^ ^15^ ^]^ Nonetheless, the binding pockets or modes of compounds A1 and A2 in hTRPV4 are entirely different from that of Trpvicin in hTRPV3‐G537S,^[^ ^15^ ^]^ suggesting that ligand‐bound induced symmetry transition may be commonly involved in the inactivation of TRPV channels.

### 2.3 Detailed Antagonist Binding Sites in hTRPV4

Although the compounds GSK279, A1, and A2 bind to similar pockets in the VSLD domain, our structures revealed that their detailed binding modes and critical interactions with hTRPV4 are different. In the case of compound A1, the aromatic residues F524 of the transmembrane segment S2, Y553 of S3, Y591, and F592 of S4 play crucial roles in sequestering the antagonist within the pocket through ring–ring stacking interactions (Figure 2B; Figure S6A, Supporting Information). Furthermore, the polar residues N474 of S1, T527 of S2, and D743 of the TRP helix, along with the nonpolar residues I744 of the TRP helix, also contribute to the binding of compound A1. To assess the contributions of these interacting residues, we generated hTRPV4 mutants within the pocket and tested their sensitivities to compound A1. The mutants showed normal activation by GSK101 and were used for further evaluation (Figures S6B and S7, Supporting Information). Specifically, the Y591A and I744A mutations reduced the potency of compound A1 by a factor of 6‐fold, as indicated by the IC_50_ values. The D743A and Y553A each caused about a 5‐fold decrease in potency. Moreover, F592A also resulted in a 3.5‐fold reduction in the potency of A1 compared to that in hTRPV4 wild‐type (hTRPV4^WT^) (Figure 2C; Figure S7 Supporting Information). These structural and functional results revealed the molecular determinants of the potent binding of compound A1 to hTRPV4.

As an analog of compound A1, compound GSK279 exhibited a similar binding pattern within the pocket, with stabilization facilitated by aromatic residues, including F524, Y553, Y591, F592, and I744, and the polar residues of N474, T527, and D743 (Figure S4F, Supporting Information). This observation implies that A1 and GSK279 employ a similar binding mode within the VSLD of hTRPV4.

The binding pose of compound A2 differs substantially from that of GSK279 or A1. The flat compound A2 lies on the TRP helix and partially sticks its two thiazole rings into the VSLD pocket. The compound is sandwiched by residues V469, S470, I473, and N474 of S1 and Y591 of S4 from the top, as well as I744 and F748 of the TRP helix from the bottom (Figure 3B). To validate these specific interactions for A2 binding, we assayed the behavior of hTRPV4 with mutations of these interacting residues in response to A2. All the tested mutants showed normal activation by GSK101 (Figure S8, Supporting Information). Compared to hTRPV4^WT^, the V469A, S470A, and I744A mutations caused increasements in the IC_50_ values of compound A2 by 4‐, 9‐, and 27‐fold, respectively (Figure 3C; Figure S8, Supporting Information), highlighting that interactions from both inside and outside the VSLD pocket are critical for A2 binding. These results support our structural observations that the large VSLD pocket can bind distinct modulators.

**Figure 4:**
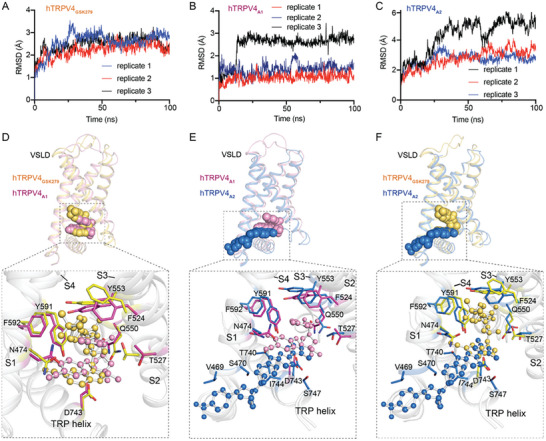
The binding differences of three antagonists in the VSLD pocket. A–C) MD simulations of GSK279, A1, and A2 binding in hTRPV4. The RMSD of antagonists is plotted against 100 ns simulation time in three independent MD trajectories. D‐F) Antagonist binding pocket comparison for hTRPV4_GSK279_ (yellow‐orange) VS hTRPV4_A1_ (pink) D), hTRPV4_A1_ (pink) VS hTRPV4_A2_ (marine) E), hTRPV4_A2_ (marine) VS hTRPV4_GSK279_ (yellow‐orange) F). The black dashed square indicates a close‐up view of the local conformational changes induced by antagonist binding for each.

To further evaluate the binding poses of the two antagonists in the VSLD revealed by the cryo‐EM structures, we conducted the MM/GBSA binding affinity calculations. The results showed that all the residues interacting with the antagonists within 5 Å contributed to the binding of the antagonist (Figures 2D and 3D), and the patterns of per‐residue contribution align with our point‐mutation functional results. For instance, Y553 and Y591 play predominant roles in A1 binding (Figure 2D), and the Y533A and Y591A mutations caused a more significant decrease in the affinity among the tested mutants (Figure 2C); moreover, both MD simulations and point mutagenesis results elucidated that V469, S470, and I744 play critical roles in A2 binding (Figure 3D,C). Furthermore, 100 ns all‐atom MD simulations were carried out on the hTRPV4 antagonist complex structures to validate the positions of the antagonists. The simulation results of three independent replicates for each antagonist revealed that compared with their original positions, GSK279 and A1 are relatively stable in the VSLD pocket, reflected by RMSD values within 3.5 Å (**Figure** **4**A,B). In contrast, A2 is more dynamic, with one replicate showing RMSD values greater than 6 Å (Figure 4C). The MD simulation results are consistent with the structural observation that GSK279 and A1 are deeply buried in the VSLD pocket, while A2 is bound to the VSLD‐extended pocket.

### 2.4 Structural Mechanisms of hTRPV4 Inhibition by the Antagonists

TRPV channel activation is complicated and tightly regulated to ensure proper cellular and physiological functions. It has been suggested that the TRP helix plays a crucial role as a structural component in the allosteric activation of the channel pore.^[^ ^19^ ^]^ It has been proposed that by binding to the VSLD pocket, antagonists can stabilize the interactions between the TRP helix and the VSLD bundles, thus inhibiting the channel; in contrast, agonists destabilize such interactions and open the pore.^[^ ^20^ ^]^ These three antagonists are not pore blockers that can physically block the pore.^[^ ^18^ ^a]^ Instead, they bind to the VSLD or the VSLD‐extended pocket. The TRP helix positions relative to the VSLD bundles are identical between the three antagonist‐bound hTRPV4 structures (Figure 4D‐F). A closer look shows that the benzimidazole ring of GSK279, the benzene ring of A1, and the thiazole ring of A2 are placed at similar positions (Figure 4D‐F), forming close interactions with the TRP helix. Although local side‐chain rotations of key interacting residues can be observed between the three structures, the TRP helix and the VSLD bundles remain highly similar. Indeed, superpositions of the hTRPV4_GSK279_ and hTRPV4_A1_ with the reported closed TRPV3 structure showed that the three antagonist‐bound hTRPV4 structures are in closed states (Figure S5, Supporting Information). Based on these structural observations, we speculate that the three antagonists employ a similar mechanism to inhibit hTRPV4; that is, the antagonist bound tightens the interactions between the TRP helix and the VSLD bundles, which prevents the opening of the intracellular gate.

### 2.5 Compounds A1 and A2 Induced the C4 to C2 Transition

**Figure 5:**
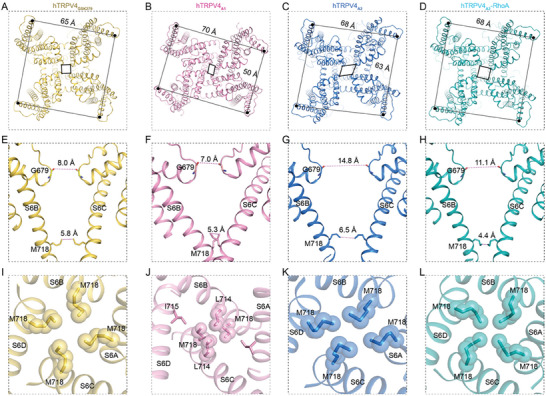
Symmetry remodeling from C4‐ to C2‐ with A1 and A2 binding. A–D) Structural rearrangements of the hTRPV4_A1_ and hTRPV4_A2_. The black dots indicate the size and shape of the transmembrane domain of each structure viewed from the top. The glycine residues at the constriction site of the SF shown as spheres indicate the conformational changes of the SF. The hTRPV4**_GSK279_** (yellow‐orange) and hTRPV4**_A2_**‐RhoA (teal) show C4‐symmetry, and the hTRPV4**_A1_** (pink) and hTRPV4**_A2_** (marine) structures are different, showing C2‐symmetry. E–H) Side view of the pore‐forming region of hTRPV4**_GSK279_**, hTRPV4**_A1_**, hTRPV4**_A2_**, and hTRPV4**_A2_**‐RhoA structures with the same color scheme as for A‐D. Only the two opposing subunits of pore domains are shown as cartoons for clarity. Diagonal distances at the SF and gate constriction points are labeled and shown with dashed lines. I–L) Bottom‐up views of the gate restrictions in hTRPV4 structures with the same color shame as for A‐D. Residues representing the helix bundle gate are displayed as sticks and spheres.

The symmetry transition of TRPV channels has been reported to be involved in channel gating.^[^ ^19^ ^]^ Ligand‐bound induced symmetry transitions have been observed in TRPV2^[^ ^14^ ^]^ and TRPV3.^[^ ^15^, ^21^ ^]^ To reveal potential symmetry transitions in hTRPV4, we performed an initial 3D classification of the EM data with C1‐symmetry imposed. The resulting EM maps of hTRPV4_A1_ and hTRPV4_A2_ showed apparent C2‐symmetry, whereas TRPV4_GSK279_ exhibited C4‐symmetry (Figures S3 and S4, Supporting Information). Because of the symmetry differences, significant conformational shifts between two neighboring subunits can be observed in the hTRPV4_A1_ and hTRPV4_A2_ structures when compared to the hTRPV4_GSK279_ structure (**Figure** **5**A‐C). Interestingly, even the twofold symmetric hTRPV4_A1_ and hTRPV4_A2_ assume distinct conformations (Figure 5B,C). The three antagonist‐bound hTRPV4 structures displayed dilated SFs with diameters of 7.0–14.8 Å and constricted inner gates with diameters of 5.3–6.5 Å (Figure 5E‐G), indicating that the inner gates of the three structures are nonconductive (Figure 5I‐K).

To analyze the conformational changes associated with symmetry changes, the hTRPV4_A1_ and hTRPV4_A2_ structures were aligned with hTRPV4_GSK279_, and the C2‐form hTRPV4_A1_ exhibited substantial structural differences from the C4‐form hTRPV4_GSK279_ (Figure S9A‐C, Supporting Information). From this overall structural comparison, it was observed that the ankyrin repeats domain (ARD) of the two opposing subunits of hTRPV4_A1_ (without A1 bound, referred to as A and C chains) exhibited an upward orientation, whereas the remaining two subunits (with A1 bound, designated as B and D chains) displayed a downwards orientation (Figure S9A, Supporting Information). In the case of hTRPV4_A2_, which shows marginal symmetry reduction, the ARD of two opposing chains (labeled as A and C chains) showed a slight downward orientation (Figure S9B, Supporting Information). To obtain more insights into the symmetry transition, we superimposed the VSLD domain of hTRPV4_A1_ and hTRPV4_A2_ with that of hTRPV4_GSK279_ (Figure S9D‐E, Supporting Information). This comparison revealed only subtle changes in the TRP helices, while significant conformational changes were observed in the pore domains. The binding of A1 and A2 to the VSLD pocket induces a local conformational change in the TRP helix and S4‐S5 linker. This, in turn, is transmitted to the pore domain and is believed to regulate the gating of TRPV4.

### 2.6 Structural Mechanisms of hTRPV4‐RhoA Interaction

During the processing of the cryo‐EM data of hTRPV4_A2_, a subset of particles showed additional strong EM densities attached to the ARDs, which was further refined to a nominal resolution of 3.44 Å with C4‐symmetry imposed (Figures S10 and S3, Supporting Information), indicating that an endogenous protein that interacts with hTRPV4 may have been copurified during the protein expression and purification processes (Figure S2B, Supporting Information). To identify which protein binds to hTRPV4, we performed a mass spectrometry analysis on the purified sample utilized for the cryo‐EM study; the results clearly showed that Ras Homolog Family Member A (RhoA) is one of the primary components present in the sample (Figure S2C, Supporting Information). We found that RhoA (PDB code: 1FTN)^[^ ^22^ ^]^ can fit perfectly in the extra EM densities (Figure S10A, Supporting Information), suggesting that endogenous RhoA binds to hTRPV4, which is consistent with the findings of previous reports on the biochemical interaction between RhoA and hTRPV4.^[^ ^23^ ^]^ The resulting hTRPV4_A2_‐RhoA structure adopts a C4 symmetric conformation (Figure 5D), which also possesses a dilated SF (11.1 Å) and a constricted inner gate (4.4 Å) (Figure 5H,L) similar to the other three hTRPV4 structures, implying that the binding of RhoA may stabilize the channel in a closed state. Structural comparison of hTRPV4‐RhoA with hTRPV4_GSK279_ showed that the ARDs underwent anticlockwise rotation upon RhoA binding (Figure S9C, Supporting Information). Some conformational differences existed in the TRP helix and S4‐S5 linker (Figure S9F, Supporting Information). The hTRPV4_A2_‐RhoA structure demonstrated that RhoA engages in extensive interactions with finger 2–4 loops of the ARD, burying a total solvent‐accessible surface area of 700 Å2 (Figure S10C,D, Supporting Information). The assembly interface of TRPV4 and RhoA involves a combination of hydrogen bonds, electrostatic interactions, hydrophobic interactions, and van der Waals interactions (Figure S10E,F, Supporting Information). More specifically, groups of polar interactions (located at Switches I and II of RhoA) play a critical role in stabilizing the inaction (Figure S10E,F, Supporting Information). In Switch II, D76 of RhoA establishes a salt bridge with R237 on finger 2 and R269 on finger 3 of hTRPV4 ARD (Figure S10E, Supporting Information). In Switch I, residues A2, R5, and E54 of RhoA form a hydrogen‐bond network with residues H265, D313, R315, and R316 from ARD fingers 3–4 of hTRPV4. Additionally, N41 of RhoA forms a hydrogen bond with the carbonyl‐oxygen of N228 of hTRPV4 (Figure S10F, Supporting Information). Sequence comparisons demonstrated that the interfacial residues present in the ARD of TRPV4 are conserved across various species, distinguishing them from the corresponding residues in other TRPV channels (Figure S6C, Supporting Information). Notably, the pathogenic mutations R232, R237 R315, and R316 have been reported to be linked to neuropathy‐causing disease,^[^ ^5^, ^23^, ^24^ ^]^ highlighting that the intrinsic interactions between TRPV4 and RhoA are critical for their normal functions and that their assembly interface is a target of pathogenic conditions. It has been implied that the interaction between TRPV4 and RhoA results in the suppression of both TRPV4 and RhoA.^[^ ^25^ ^]^ The TRPV4‐RhoA complex is the basis for comprehending the structural interplay between these significant proteins. Based on the structural data obtained from the TRPV4‐RhoA interface in the channelopathies, we suggest that this interface can be exploited to discover molecule glue^[^ ^26^ ^]^ as a therapeutic for treating related diseases.^[^ ^27^ ^]^ In addition to channelopathies, it has been found that TRPV4 plays a role in regulating angiogenesis through the modulation of endothelial cell mechanosensitivity via the Rho/Rho kinase pathway and can serve as a potential therapeutic target for cancer therapy.^[^ ^25^ ^a]^ The residues R5 and Y42 of RhoA are situated at the TRPV4‐RhoA interface. These residues, which were found to be commonly altered in 30 diffuse‐type gastric carcinomas (DGC) cases through whole‐exome sequencing, are highly conserved among RHO family proteins.^[^ ^28^ ^]^ Further investigations are needed to determine the direct correlation between these mutations and their impact on TRPV4 modulation and associated effects.

In preparing the manuscript of this study, Kwon et al. and Nadezhdin et al. reported similar structures of TRPV4 in complex with GSK279^[^ ^13^ ^a]^ and the TRPV4‐RhoA complex,^[^ ^13^ ^a,^ ^13^ ^b]^ together with the structural observations of our study, these findings provide important insights into the intrinsic interactions between TRPV4 and RhoA, pathogenic mutations that disrupt these interactions, and potential regulatory mechanisms of their functions.

## 3 Conclusion

This study presented the cryo‐EM structures of hTRPV4 in a complex with two potent small‐molecule antagonists, and the patch‐clamp assay verified the vital residues responsible for their binding. This study further demonstrated the versatility and adaptability of the VSLD pocket, which can accommodate ligands with diverse backbones. The A2 binding position may constitute a novel binding mode. The transition from C4‐ to C2‐ symmetry in TRP channel gating was detected during ligand‐induced gating in TRPV2,^[^ ^29^ ^]^ TRPM2,^[^ ^30^ ^]^ and TRPV3^[^ ^21^ ^]^ channels. The hTRPV4_A1_ and hTRPV4_A2_ structures exhibited a noteworthy change in symmetry, which was not previously observed in TRPV4. These findings suggest that C4‐ to C2‐symmetric conformational changes may constitute a shared mechanism within the TRP channel family^[^ ^31^ ^]^ that is regulated by the fine‐tuning of the S4‐S5 linker and TRP helix, resembling a gearbox mechanism.^[^ ^32^ ^]^ Overall, the structures and the modulation mechanisms of TRPV4 have important implications for both fundamental and translational research.

## 4 Experimental Section

### Compounds

GSK2798745 (GSK279, MedChemExpress, HY‐19765), GSK3527497 (A1, ProbChem, PC‐73096) and GSK1016790A (GSK101, MedChemExpress, HY‐19608) were purchased from the vendors. The antagonist A2 was synthesized (Note S1, Supporting Information).

### Plasmid Constructs

The full‐length cDNA of hTRPV4 (NM_001177431.1) and mTRPV4 (XM_006530432.2) was amplified from the HEK293T cDNA and mouse cDNA library; Then the genes were cloned into pEG BacMam vector fused with a GFP‐twin‐Strep tag at the C‐terminus for expression and functional evaluation. All hTRPV4 mutants were generated by site‐directed mutagenesis. The primer information was included in Supporting Information Table S1 (Supporting Information). All constructs used in this study were confirmed by DNA sequencing.

### Electrophysiology

The Chinese hamster ovary (CHO) cells were cultured in Dulbecco's Modified Eagle Medium (DMEM, Gibco, USA) and Ham's F‐12 Nutrient Mixture (F‐12, Gibco, USA) mixed with a proportion of 1:1 and supplemented with 10% (v/v) Fetal Bovine Serum (FBS, PAN‐Biotech, Germany) at 37 °C and 5% CO_2_. Corresponding plasmids were transfected using Lipofectamine 2000 following the manufacturer's instructions (Invitrogen). Electrophysiological experiments were performed 13–24 h after transfection. Whole‐cell patch‐clamp recordings were obtained using a HEKA EPC‐10 patch‐clamp amplifier (HEKA Electronic) and PatchMaster software (HEKA Electronic). The pipettes were fabricated by a DMZ Universal Electrode puller (Zeitz Instruments) using borosilicate glass, with a resistance of 2–4 MΩ for whole‐cell patch clamp recordings. An Ag‐AgCl wire was employed as a reference electrode, and signals were filtered at 5 kHz. Cell membrane capacitances were monitored and used to calculate current densities. Membrane potential was held at 0 mV. For recording agonist‐induced activation, currents were elicited by a protocol consisting of a 400‐ms step to +60 mV, followed by a 400‐ms step to −60 mV at 2‐s intervals. For the recording of inhibition by A1 or A2, currents were elicited by a protocol consisting of a 400‐ms step to +60 mV. For the recording of TRPV4 channels, pipette solutions contained 140 mm CsF, 1 mm MgCl_2_, 10 mm HEPES, 0.1 mm CaCl_2_ and 5 mm EGTA (buffered to pH 7.3 with CsOH), and bath solutions contained 140 mm NaCl, 5 mm KCl, 1 mm MgCl_2_, 10 mm D‐glucose, 10 mm HEPES and 2 mm CaCl_2_ (buffered to pH 7.3 with NaOH). All recordings were made at room temperature.

### Primary Skin Keratinocyte Isolation

Three wild‐type and mTRPV4 knockout (mTRPV4KO) mice (male, 8–9 weeks) were used to prepare keratinocytes. Tails were dissected and incubated with 4 mg mL^−1^ Dispase (Wako,383‐02281) overnight at 4 °C. After 16 h of incubation; the epidermis was separated from the dermis followed with 0.25% trypsin (Gibco, 15050065) treatment for 20 min at room temperature. Primary skin keratinocytes were next dissociated by forceps and seeded on the coverslips with incubation at 37 °C in 5% CO_2_. The MCDB153 culture medium (CSR, CK105) containing 5 µg mL^−1^ insulin (Sigma, I1882), 0.4 µg mL^−1^ hydrocortisone (Sigma, H0888), 10 µg mL^−1^ transferrin (SCIPAC, P158‐5), 14.1 µg mL^−1^ phosphorylethanolamine (Sigma, P0503), 10 ng mL^−1^ epidermal growth factor (Sigma, E4127), 25 µg mL^−1^ gentamicin (Gibco, 15710064), 50 units per mL penicillin, 50 µ mL^−1^ streptomycin, and 40 µg mL^−1^ bovine pituitary extracts (Kyokuto, 20200) was used.

### Whole‐Cell Patch‐Clamp on Keratinocytes

All the recordings were performed on days 3–4 after cell isolation. The currents were recorded from −100 to +100 mV voltage ramps every 5 s with a −60 mV holding potential, by applying 300 nm GSK101 (Sigma) with/without A1 or A2 to the bath solution. The extracellular bath solution contains 140 mm NaCl, 5 mm KCl, 2 mm MgCl_2_, 10 mm glucose, 5 mm EGTA, and 10 mm HEPES, pH 7.4 adjusted with NaOH. The pipette solution contains 140 mm KCl, 5 mm EGTA, and 10 mm HEPES, pH 7.4 adjusted with KOH. The pipette with a resistance of 4–5 MΩ was used. The data were recorded at 10 kHz (pCLAMP) and filtered at 5 kHz (Clampfit). *I*–*V* curves were plotted with Prism 10.

### Ca^2+^ Imaging on Keratinocytes

All the recordings were performed on days 2–3 after cell isolation. Before recordings, 5 µm fura‐2‐acetoxymethyl ester (Fura2‐AM, Invitrogen, F1201) was incubated for 1 h. A series of A1 or A2 with different concentrations were prepared in the bath solution (140 mm NaCl, 5 mm KCl, 2 mm CaCl_2_, 2 mm MgCl_2_, 10 mm glucose, and 10 mm HEPES, pH 7.4 adjusted with NaOH) containing 300 nm GSK101. During recordings, the Fura2 was excited at 340 and 380 nm, and the 340/380 nm ratio indicated the intracellular Ca^2+^ changes upon each stimulation. The data were obtained and analyzed using NIS‐Elements AR (Nikon), Microsoft Excel, and Prism 10.

### Protein Expression and Purification

The expression and purification protocol for hTRPV4 was similar to what hTRPV3 was used for.^[^ ^15^ ^]^ The recombinant baculovirus was generated by insect Sf9 cells. P2 viruses infect HEK293F GnTI^−^ cells at 2×10^6^/mL cells. After 8–10 h post‐infection, 10 mM sodium butyrate was added to boost protein expression. Cells were harvested 48 h later by centrifugation at 1800 g and were frozen at −80 °C until further purification. The whole purification process was either carried out on the ice or at 4 °C. Cells were resuspended in TBS buffer (20 mm Tris, pH 8.0, 150 mm NaCl) in the presence of protease inhibitors including 250 µm phenylmethylsulfonyl fluoride (PMSF), 0.8 µm Aprotinin, 4.7 µm Leupeptin, 2 µm Pepstatin A. Dounce homogenizer was used for cells disruption and then membrane debris was collected by ultracentrifugation for 1 h (Beckman, Ti32 rotor, 100 000 g). The membrane fraction was homogenized and resuspended in TBS buffer supplemented with 1% (w/v) dodecyl‐β‐d‐maltopyranoside (DDM) (Jiejing Tech Inc., China) plus 0.2% CHS (Anatrace, USA) and was slowly stirred for 2 h. The supernatant was collected by another 40 min of ultra‐centrifugation and passed through a 0.22 µm filter before loading onto the gravitate column packed with Streptactin Beads (Smart‐Lifesciences, China). The resin was then washed with ten column volumes of TBS supplemented with 0.06% (w/v) GDN (Anatrace, USA). hTRPV4 was eluted with TBS containing 0.06% (w/v) GDN and 5 mm desthiobiotin (Sigma, USA). The eluted sample was concentrated and loaded onto a Superose 6 column (GE Healthcare, USA) equilibrated with TBS plus 0.007% (w/v) GDN. Peak fractions corresponding to the tetrameric hTRPV4 were pooled and concentrated to ≈10 mg mL^−1^ for cryo‐EM sample preparation. The purification processes were supplemented with 10 µm antagonist and an additional 100 µm antagonist for GSK279, A1, and A2 was added to the final concentrated protein sample respectively.

### Cryo‐EM Grid Preparation and Data Collection

The purified 3 µL hTRPV4 with an antagonist was applied onto a glow‐discharged Quantifoil holey carbon grid (copper, Quantifoil R1.2/1.3, Germany). The grid was then blotted for 2.0–4.0 s at 4 °C and 100% humidity using Vitrobot Mark IV (Thermo Fisher Scientific, USA), and then was plunged into liquid ethane cooled by liquid nitrogen. Data were collected on the FEI Titan Krios (Thermo Fisher Scientific, USA) electron microscope operating at 300 kV using a K2 Summit direct electron detector positioned after a GIF quantum energy filter (Gatan, USA). Raw movies were automatically acquired in super‐resolution mode with a physical pixel size of 1.04 Å for the K2 detector using SerialEM. Every movie stack was recorded for 6.4 s fractionated into 32 frames with a total accumulated dose of ≈60 e Å^−2^ According to the available Cryo‐EM time, a total of 1830, 2601, and 4118 movie stacks were collected for hTRPV4_GSK279_, hTRPV4_A1_, and hTRPV4_A2_.

### Image Processing

Using MotionCorr2,^[^ ^33^ ^]^ all the movie stacks were motion‐corrected, binned twofold, and dose‐weighted. Gctf^[^ ^34^ ^]^ was applied to estimate the defocus values of the resulting summed micrographs. Template particle picking, 2D and 3D classification, particle polishing, and CTF refinement were carried out in Relion3.^[^ ^35^ ^]^ To reveal possible symmetry differences in these structures, C1 symmetry was used for all global 3D classifications in Relion3. Subsequently, for local 3D classification and non‐uniform refinement, C4 symmetry was applied to hTRPV4_GSK279_ and hTRPV4_A2_‐RhoA which exhibited C4 symmetry; and C2 symmetry was utilized for hTRPV4_A1_ and hTRPV4_A2_ which demonstrated C2 symmetry. The final best class contained 206979, 102087, 48492, and 47803 particles imported in cryoSPARC^[^ ^36^ ^]^ and were refined to 3.45, 3.74, 3.21, and 3.44 Å for hTRPV4_GSK279_, hTRPV4_A1_, hTRPV4_A2,_ and hTRPV4_A2_‐RhoA respectively. Detailed data processing diagrams were presented in Supporting Information Figures S3 and S4 (Supporting Information).

### Model Building

The structure of hTRPV3 (PDB code:7xj0) was fitted in the cryo‐EM density map of hTRPV4_A1_ using Chimera.^[^ ^37^ ^]^ For the structures of hTRPV4_A1_, hTRPV4_A2_, and hTRPV4_A2_‐RhoA, the hTRPV4_GSK279_ was used as the initial model. The fitted models were manually inspected and corrected in COOT^[^ ^38^ ^]^ and subsequently refined using Phenix^[^ ^39^ ^]^ real‐space refinement. Statistics for cryo‐EM data collection and model refinement are summarized in Table S2 (Supporting Information). All figures were prepared with PyMOL (Schrödinger, LLC)^[^ ^40^ ^]^ and ChimeraX.^[^ ^41^ ^]^

### Trypsin Digestion and MS Analysis

20 µg of purified hTRPV4 protein was precipitated with a fourfold volume of acetone for at least 30 min at −20 ˚C. The pellets obtained were air‐dried and dissolved, assisted by sonication in a solution of 8 m urea, 20 mm methylamine, and 100 mm Tris (pH 8.5). Reduction and alkylation of the sample were carried out using 5 mm TCEP for 20 min at 25  C, followed by 10 mm iodoacetamide for 15 min in the dark. The samples were then diluted to 2 m urea with 100 mm Tris (pH 8.5). Denatured proteins were digested by trypsin at a 1/50 (w/w) enzyme/substrate ratio at 37 °C for 16–18 h, and the reactions were quenched with 5% formic acid (final conc.). The LC−MS/MS analysis was performed using Easy‐nLC 1000 II HPLC system (Thermo Fisher Scientific) coupled with the Q‐Exactive plus mass spectrometer (Thermo Fisher Scientific). Peptides were loaded on a pre‐column and separated on a homemade analytical column (75 µm × 12 cm). Peptides were injected and separated with a 75 min linear gradient at a flow rate of 300 nL min^−1^ as follows: 2–6% B in 2 min, 6–44% B in 60 min, 44–100% B in 4 min, 100% B for 9 min (A = 0.1% FA, B = 80% ACN, 0.1% FA). The top fifteen most intense precursor ions from each full scan (resolution 70 000) were isolated for HCD MS2 (resolution 17500; NCE 28) with a dynamic exclusion time of 40 s. Precursors with 1+, 8+, more than 8+, or unassigned charge states were excluded. Proteins were identified by Thermo Proteome Discoverer 2.4. The search parameters used for Proteome Discoverer were as follows, Sequest HT: protein database: homo‐sapiens; enzyme name: trypsin; max. Missed cleavage sites: 2; min. Peptide length: 6; max. Peptide length: 144; precursor mass tolerances: 10 ppm; fragment mass tolerances: 0.02 Da; static modification: carbamidomethyl/ + 57.021 Da; The results were filtered by PSM false identification rate (strict) < 0.01.

### All‐Atom Molecular Dynamics Simulations

CHARMM‐GUI generated all the protein‐ligand bilayer membrane systems. The POPC was used to build the phospholipid bilayer. The concentration of NaCl was set to 0.15 m. The ff19SB force field parameter set was used for the protein, the gaff2 force field parameter set for ligands, the TIP3P model for water and the lipid21 force field parameter set for the lipid. Long‐range electrostatic effects were modeled using the particle mesh Ewald method with periodic boundary conditions. An 8 Å cut‐off was applied to Lennard‐Jones and electrostatic interactions. For the system, three independent molecular dynamics simulations were performed according to the following steps: 1) Minimization for waters and lipids was performed with a maximum cycle of 10 000 and with the steepest descent algorithm for the first 5000 cycles with the SHAKE algorithm (an algorithm for constrained molecular dynamics) inactivated. Positional restraints of 0.5 kcal mol^−1^Å^−2^ were applied to the protein, ligands, and phospholipid. 2) Minimization for the whole system was performed with a maximum cycle of 10 000 and the steepest descent algorithm for the first 5000 cycles with the SHAKE algorithm inactivated. 3) A 1 ns Heating process was performed with a periodic boundary for constant volume with the SHAKE algorithm activated. Thus, the angle between the hydrogen atoms was fixed. The temperature increased from 0 to 303 K with Langevin dynamics with a collision frequency of 2 ps^−1^. 4) A 1 ns density equilibrium process was performed with a periodic boundary for constant pressure with anisotropic pressure scaling and a constant temperature of 303 K. 5) A 100 ns equilibrium process was performed with a periodic boundary for constant pressure and constant temperature of 303 K. Three independent dynamic simulations were performed for each complex structure. The analysis of the trajectory was done by Chimera1.15.^[^ ^37^ ^]^

### MM/GBSA Binding Free Energy Calculation

Trajectory sampled by MD simulations were used for MM/GBSA binding affinity calculations and decompositions in the Amber20^[^ ^42^ ^]^ program. All reported binding affinities and the decomposed components were averaged over 3 independent 100 ns MD trajectories (1 sample per ns). Entropic contributions were not calculated.

## Conflict of Interest

The authors declare no conflict of interest.

## Author Contributions

J.F. and C.G. contributed equally to this work. X.L. initiated and supervised the project in collaboration with Z.H. and J.F. J.F. performed sample preparation, data acquisition, and structure determination with the help of W.X. The patch clamp experiment with a heterologous expression system was performed by C.G. under Z.H.’s supervision. The patch clamp experiment with primary keratinocyte cells was performed by J.L. under M.T.’s supervision. Compound synthesis was carried out by D.L. H.K. conducted the MM/GBSA calculations and molecule dynamic simulations. J.F. and Y.T. conducted the Mass Spectrometry experiment. J.F. wrote the manuscript with input from all the authors.

## Supporting information

Supporting Information

## Data Availability

The cryo‐EM maps for hTRPV4_GSK279_, hTRPV4_A1_, hTRPV4_A2_, and hTRPV4_A2_‐RhoA have been deposited in the eceltron microscopy dat bank with accession codes EMD‐36660, EMD‐36659, EMD‐36675, and EMD‐36676. Atomic coordinates for hTRPV4_GSK279_, hTRPV4_A1_, hTRPV4_A2_, and hTRPV4_A2_‐RhoA have been deposited in the protein data bank under accession codes 8JU6, 8JU5, 8JVI, and 8JVJ.
