# Supplementary material for: Structural Pharmacology of TRPV4 Antagonists
Source: Adv Sci (Weinh). 2024 Apr 24;11(25):2401583. doi: 10.1002/advs.202401583 (PMC11220649; doi:10.1002/advs.202401583)
Supplement: Supplementary file 1 — Supporting Information [file ADVS-11-2401583-s001.pdf]

ADVANCED SCIENCE
Open Access

Supporting Information

for *Adv. Sci.*, DOI 10.1002/advs.202401583

Structural Pharmacology of TRPV4 Antagonists

Junping Fan, Chang Guo, Daohong Liao, Han Ke, Jing Lei, Wenjun Xie, Yuliang Tang, Makoto Tominaga, Zhuo Huang* and Xiaoguang Lei**

Supporting Information

Structural Pharmacology of TRPV4 Antagonists

Junping Fan,# Chang Guo,# Daohong Liao, Han Ke, Jing Lei, Wenjun Xie, Yuliang Tang, Makoto Tominaga, Zhuo Huang,* and Xiaoguang Lei**

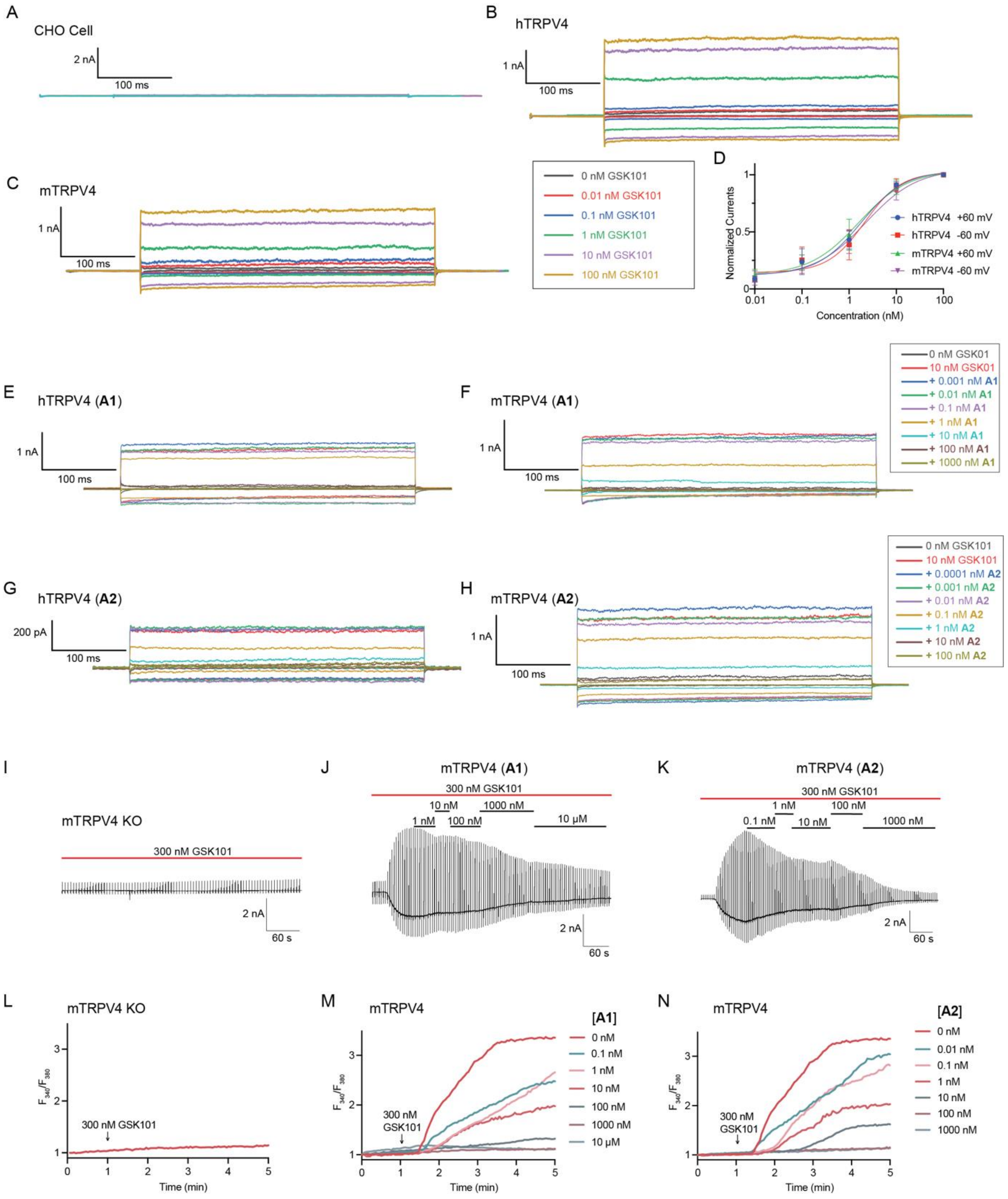

Figure S1. The current traces and statistics of TRPV4 activated by GSK101 and inhibited by A1 and A2. A-C) The representative current traces of blank (A), hTRPV4 transfected (B), and mTRPV4 transfected (C) CHO cell activated by GSK101. D) Curve fitting of dose-dependent activation of TRPV4 by GSK101 at ±60 mv (+60 mv hTRPV4 EC_{50} = 1.78 ± 0.44 nM, n = 4; -60 mv hTRPV4 EC_{50} = 2.14 ± 0.64 nM, n = 4; +60 mv mTRPV4 EC_{50} = 1.59 ± 0.45 nM, n = 4; -60 mv mTRPV4 EC_{50} = 2.29 ± 0.66 nM, n = 4). E-F) The representative current traces of hTRPV4 (E), and mTRPV4 (F) 's inhibition by A1 at ±60 mv evoked by 10 nM GSK101, (+, co-application of 10 nM GSK101). G-H) The representative current traces of hTRPV4 (G), and

mTRPV4 (H) 's inhibition by A2 at ±60 mv evoked by 10 nM GSK101 (+, co-application of 10 nM GSK101). I) A representative current trace induced by 300 nM GSK101 in keratinocytes lacking mTRPV4 (mTRPV4KO). J-K) Representative current traces activated by 300 nM GSK101 and inhibited by A1 (J) or A2 (K) at different concentration gradients in keratinocytes from wild-type mice. L) Representative changes of intracellular Ca^{2+} induced by 300 nM GSK101 only in primary skin keratinocytes from mTRPV4KO. M-N) Representative changes of intracellular Ca^{2+} induced by 300 nM GSK101 with different concentrations of A1 (M) or A2 (N) in primary skin keratinocytes from wild-type mice (arrow, 300 nM GSK101 added).

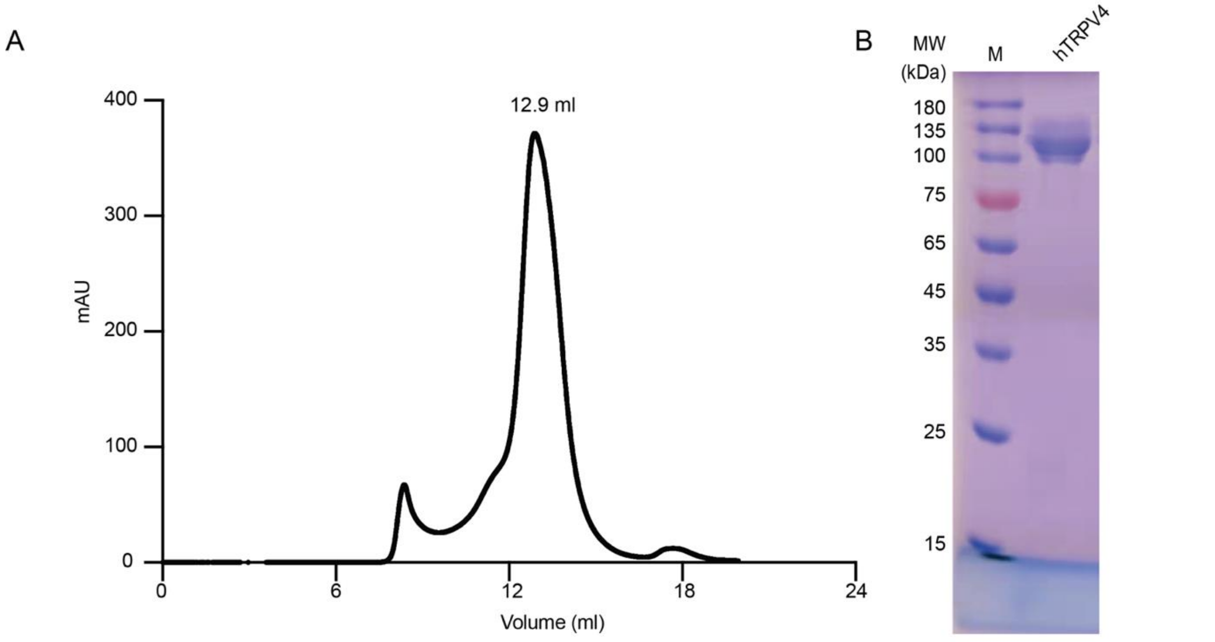

C

Accession	Description	Coverage [%]	# Peptides	# PSMs	# Unique Peptides	# AAs	MW [kDa]
Q5JR08	RhoC	88	19	185	8	188	21.5
P61586	RhoA	87	20	258	10	193	21.8
P62745	RhoB	84	19	146	16	196	22.1
P0DP25	Calmodulin-3	79	11	48	11	149	16.8
Q9HBA0	TRPV4	73	82	1709	82	871	98.2

Figure S2. Purification and mass spectrometry of hTRPV4. A) Representative gel-filtration profile of purified hTRPV4 run with Superose 6 Increase 10/30 column. The peak corresponding to target hTRPV4 at 12.9 ml was labeled. B) The eluted peak used for cryo-EM grid preparation was shown on sodium dodecyl sulfate-polyacrylamide gel electrophoresis (SDS-PAGE) with Coomassie staining. Representative figures from three independent experiments were shown. C) List of the major proteins identified by mass spectrometry from the sample used for Cryo-EM sample preparation.

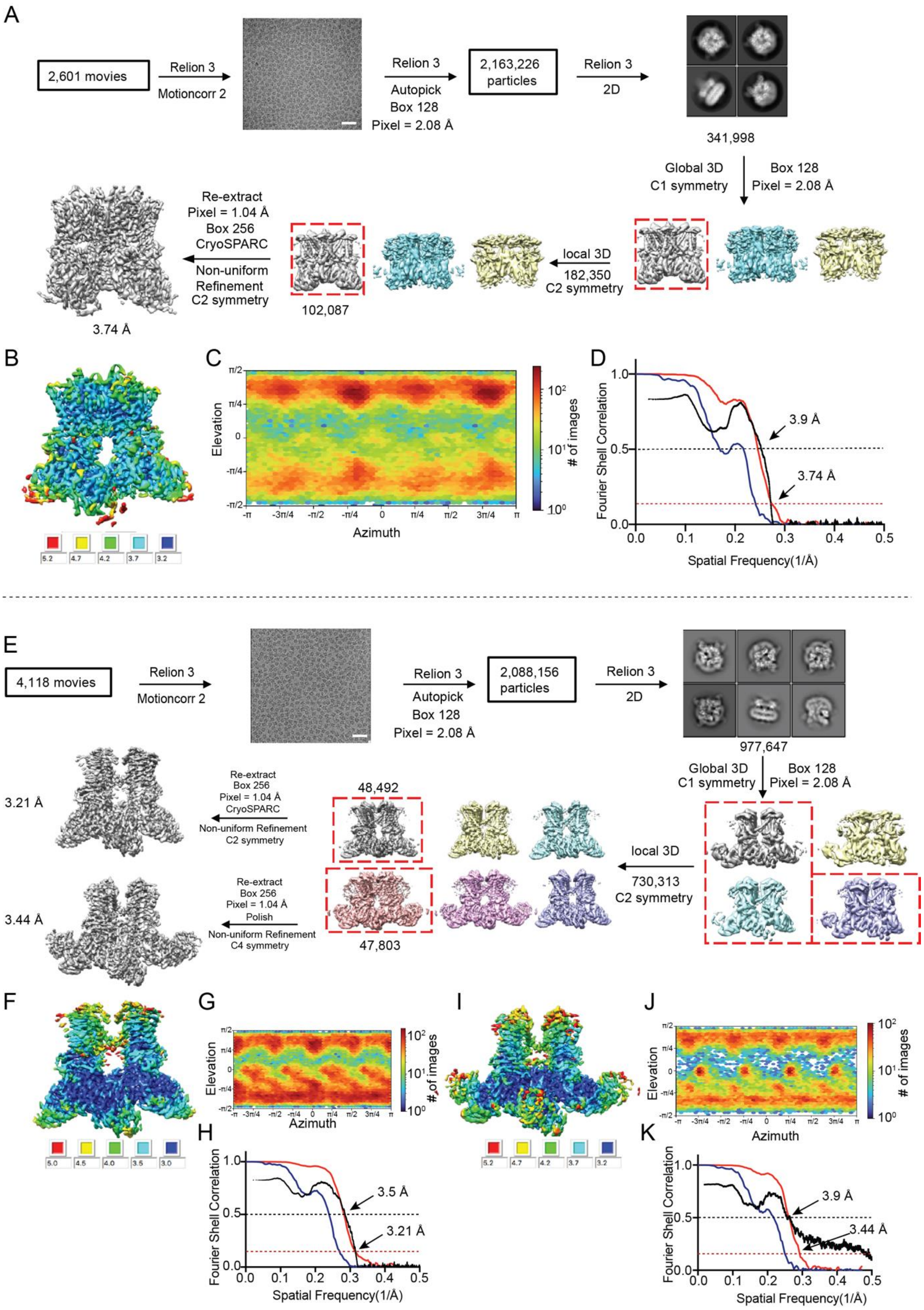

Figure S3. Cryo-EM data processing of hTRPV4 in complex with antagonists A1 and A2. A) Flow chart of cryo-EM data processing for $hTRPV4_{A1}$. 2,163,226 particles were picked from 2,601 micrographs. A representative motion-corrected micrograph of the dataset is shown here

(Bar = 500 Å). To clean particles, several rounds of 2D and 3D classifications were conducted. Then Bayesian Polish and CTF Refine were applied for improving the image quality. According to the GSFSC criterion, the final map was refined at 3.74 Å. B) The sharpened map of the $hTRPV4_{A1}$ with local resolution distributed. C) Particle angular distribution for the final reconstruction of $hTRPV4_{A1}$ calculated in cryoSPARC. D) Fourier Shell Correlations (FSC) of the final map of the $hTRPV4_{A1}$, calculated between two independently refined half-maps before and after post-processing shown in blue and red respectively, overlaid with an FSC curve calculated between the cryo-EM density map and the structural model shown in black.

E) Flow chart of cryo-EM data processing for $hTRPV4_{A2}$. 4,118 micrographs were collected and a total of 2,088,156 particles were picked. A representative motion-corrected micrograph of the dataset is shown (Bar = 500 Å). Several rounds of 2D and 3D classifications were conducted to clean particles, followed by Bayesian Polish and CTF Refine to improve image quality. The final map was refined at 3.21 Å and 3.44 Å for $hTRPV4_{A2}$ and $hTRPV4_{A2}$-RhoA according to the GSFSC criterion, respectively. (F&I) Local resolution distribution of the sharpened map of the $hTRPV4_{A2}$ and $hTRPV4_{A2}$-RhoA. (G&J) Particle angular distribution calculated in cryoSPARC for the final reconstruction of $hTRPV4_{A2}$ and $hTRPV4_{A2}$-RhoA. (H&K) FSC of the final map of $hTRPV4_{A2}$ and $hTRPV4_{A2}$-RhoA, calculated between two independently refined half-maps before (blue) and after (red) post-processing, overlaid with an FSC curve calculated between the cryo-EM density map and the structural model shown in black.

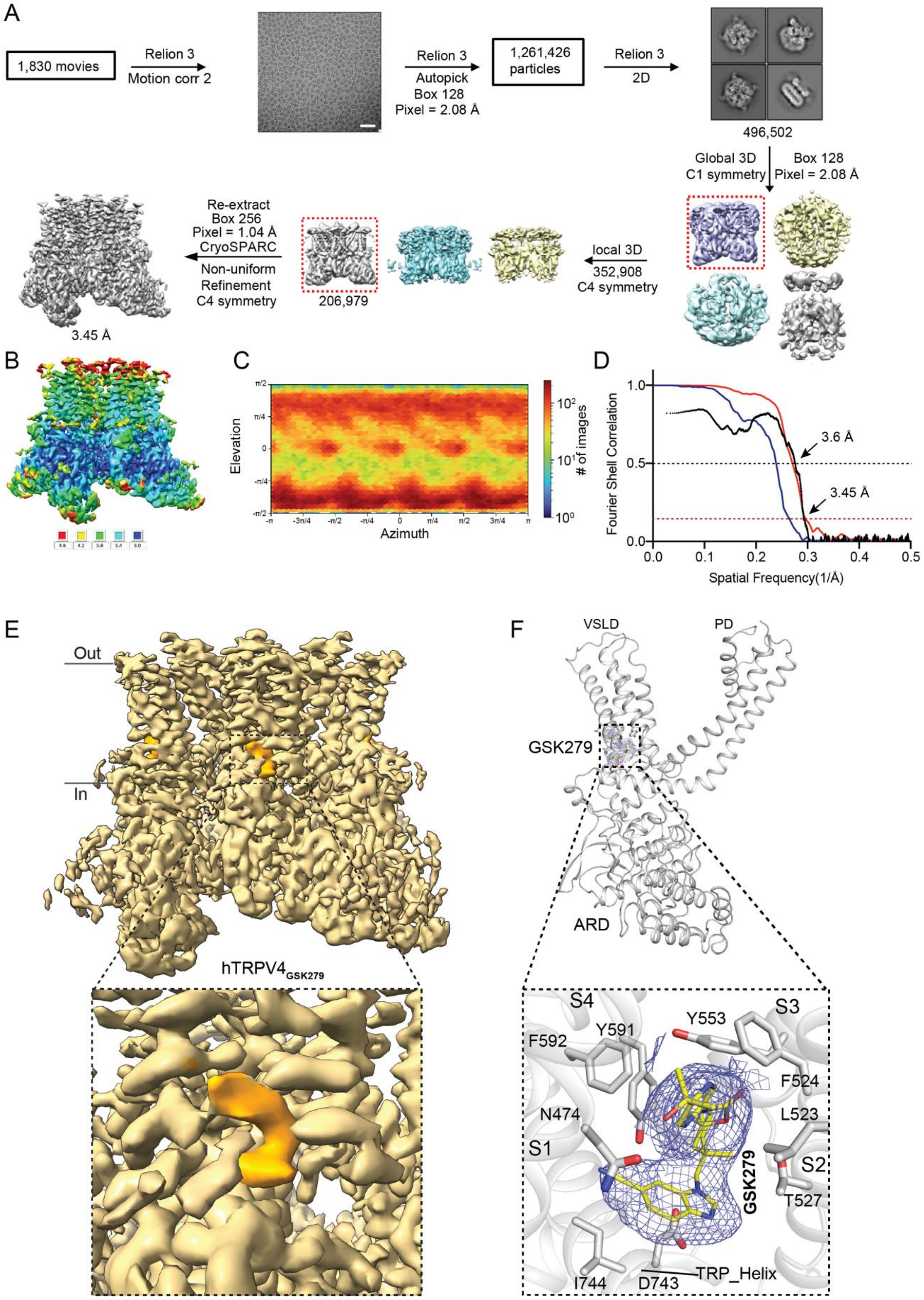

Figure S4. Cryo-EM data processing of $hTRPV4_{GSK279}$ and detailed binding sites for GSK279 in hTRPV4. A) Flow chart of cryo-EM data processing. Totally 1,261,426 particles were picked from 1,830 micrographs, with a representative motion-corrected micrograph of the dataset shown here (Bar = 500 Å). After several rounds of 2D and 3D classifications conducted to clean particles, Bayesian Polish and CTF Refine were applied to improve image quality. According

to the GSFSC criterion, the final map was refined at 3.45 Å. B) The sharpened map of the $hTRPV4_{GSK279}$ with local resolution distribution. C) Particle angular distribution for the final reconstruction of $hTRPV4_{GSK279}$ calculated with cryoSPARC. D) $hTRPV4_{GSK279}$ final map's FSC calculated between two independently refined half-maps before (blue) and after (red) post-processing, overlaid with an FSC curve calculated between the cryo-EM density map and the structural model (black). E) Cryo-EM reconstruction of hTRPV4 in the GSK279-bound form. The black dashed square indicates the region shown in enlarged views to provide the detailed binding pocket for GSK279. The electron density of the antagonists inside the VSLD pocket is highlighted for clarity. F) A single chain of $hTRPV4_{GSK279}$ viewed parallel to the membrane. On the bottom are the close-up views of the detailed interactions between hTRPV4 and GSK279. Antagonists are shown in sticks and colored with carbon atoms in yellow, oxygen in red, and nitrogen in blue. The density around the antagonist is contoured at 5 σ (blue mesh). Transmembrane segments S1-S4 and TRP helix are colored in grey. Side chains of key residues interacting with antagonists are shown in the sticks.

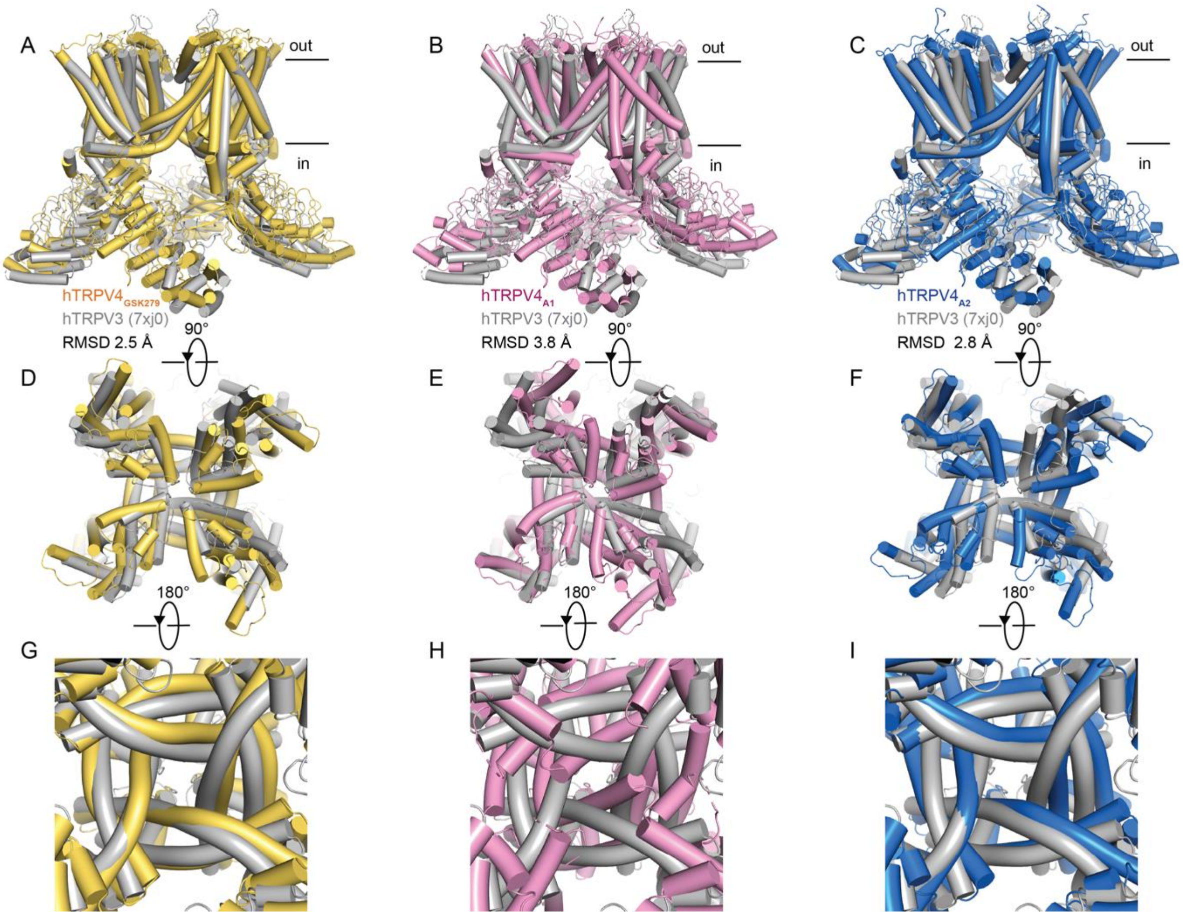

Figure S5. Structure comparisons between hTRPV4 and hTRPV3. A-C) All the structures are shown as cartoon, with hTRPV3 (7xj0) colored in grey, $hTRPV4_{GSK279}$ in yellow-orange, $hTRPV4_{A1}$ in pink, and $hTRPV4_{A2}$ in marine. The overall conformational difference is viewed

parallel to the membrane for hTRPV4$_{GSK279}$ VS hTRPV3$_{Trpvicin}$ (A), hTRPV4$_{A1}$ VS hTRPV3$_{Trpvicin}$ (B), and hTRPV4$_{A2}$ VS hTRPV3$_{Trpvicin}$ (C). D-F) The overall conformational difference is viewed from the top. G-I) The overall conformational difference is viewed from the bottom.

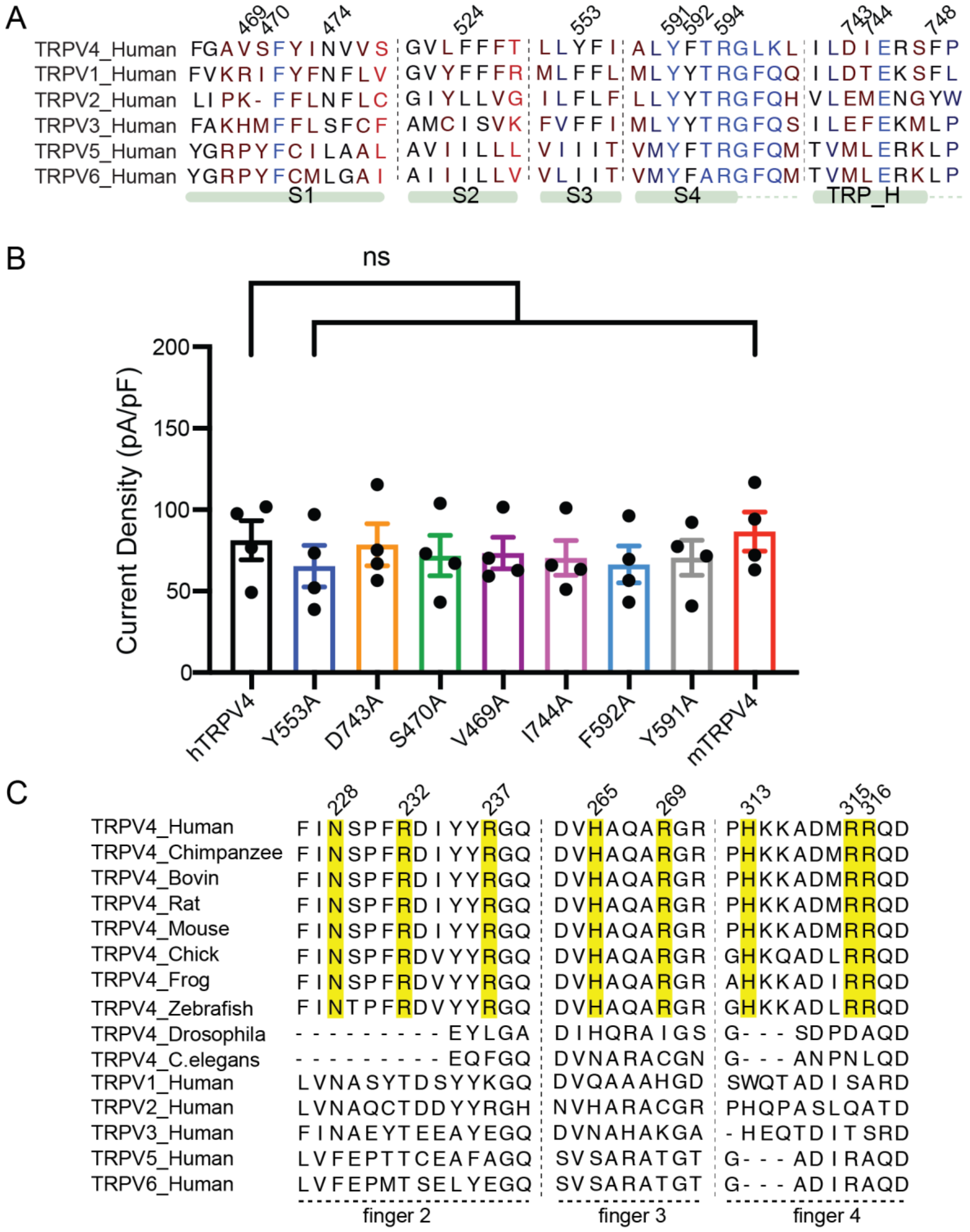

Figure S6. Sequence alignments and evaluations of the binding and interacting sites. A) Sequence alignment of the human TRPV channels for the antagonists' binding sites. The residues are colored based on sequence similarity, with high conserved in blue and less

conserved in red. Residues contributed to GSK279, A1, and A2 binding are labeled. B) The hTRPV4 mutants function similarly to the hTRPV4^{WT} in response to 10 nM GSK101. The current density at ±60 mV of hTRPV4 mutant channels activated by 10 nM GSK101, compared with hTRPV4^{WT}. C) Sequence alignment of the TRPV4 channels from various species with human TRPV channels for the RhoA binding sites. The conserved residues are highlighted with a yellow background.

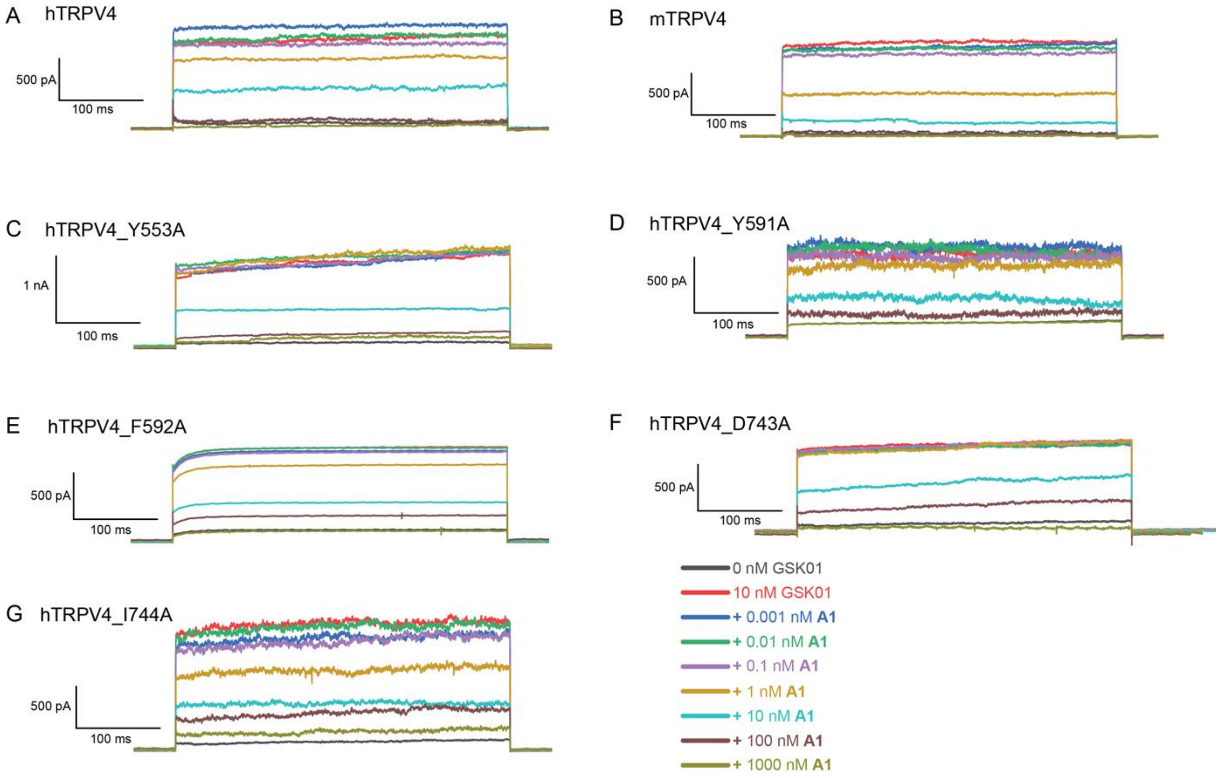

Figure S7. The current traces of TRPV4 and mutations at A1 binding sites. A-G) The representative current traces of hTRPV4 (A), mTRPV4 (B), and hTRPV4 mutations (C-G) in the A1 VSLD binding sites in response to 10 nM GSK101 and co-application of increasing concentrations of A1 at +60 mV (+, co-application of 10 nM GSK101).

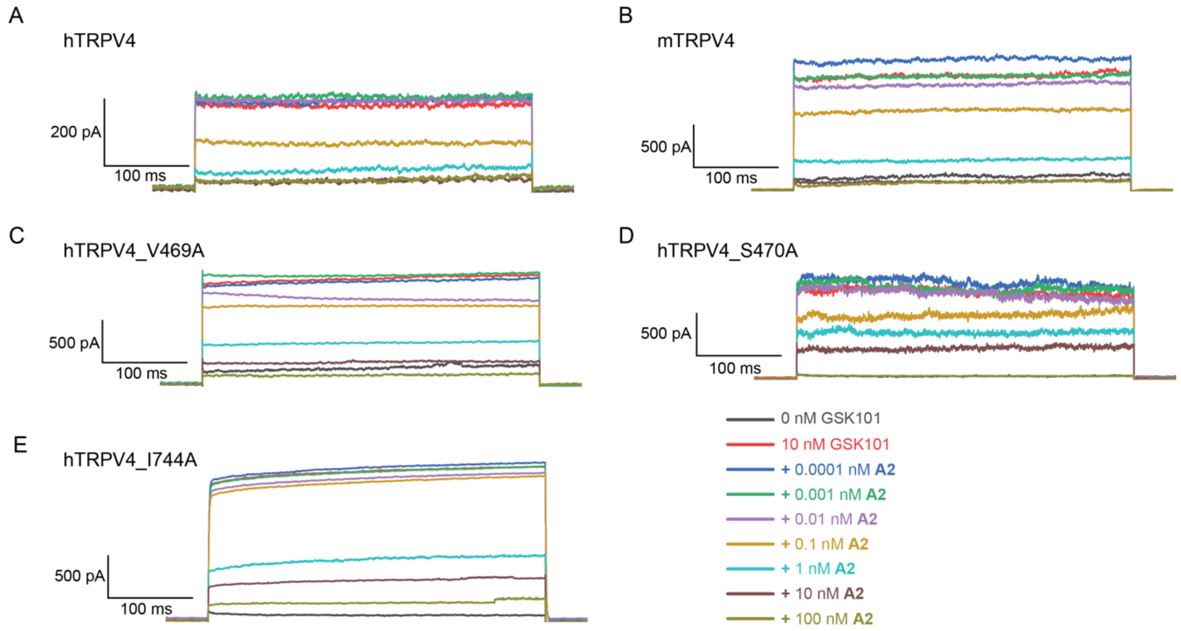

Figure S8. The current traces of TRPV4 and mutations at A2 binding sites. A-E) The representative current traces of hTRPV4 (A), mTRPV4 (B), and hTRPV4 mutations (C-E) in the A2 VSLD binding sites in response to 10 nM GSK101 and co-application of increasing concentrations of A2 at +60 mV (+, co-application of 10 nM GSK101).

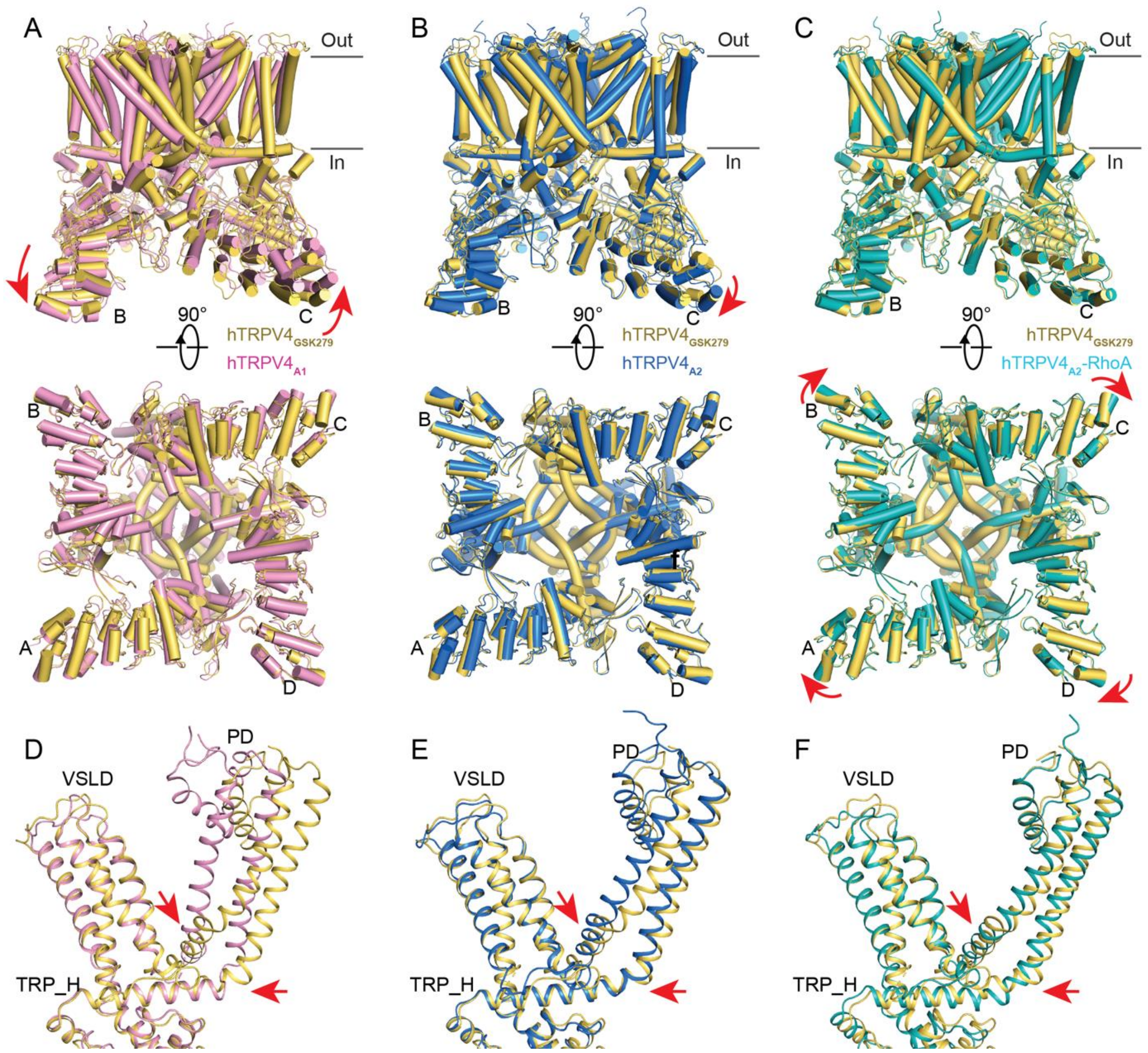

Figure S9. Conformational changes of antagonists and RhoA binding in hTRPV4. A-C) Superposition of the $hTRPV4_{A1}$ (A) $hTRPV4_{A2}$ (B) and $hTRPV4_{A2}$-RhoA (C) with $hTRPV4_{GSK279}$ viewed parallel to the membrane (top) and intracellularly (bottom). D-F) Superposition of the TM domain $hTRPV4_{A1}$ (D) and $hTRPV4_{A2}$ (E) and $hTRPV4_{A2}$-RhoA (F) with $hTRPV4_{GSK279}$ viewed parallel to the membrane. Arrows indicate conformational changes.

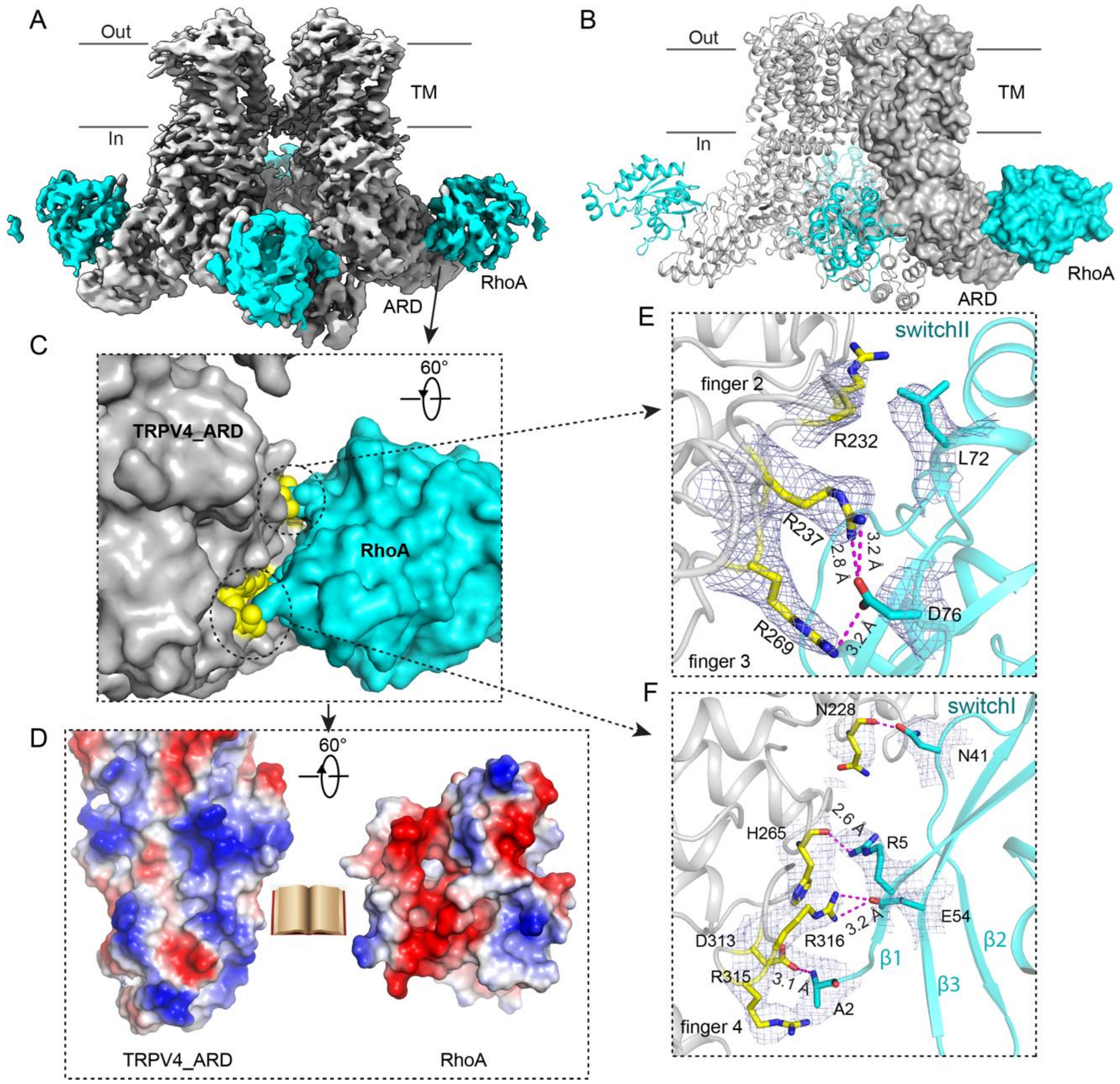

Figure S10. The complex structure of hTRPV4$_{A2}$-RhoA. A) 3D reconstruction of hTRPV4$_{A2}$-RhoA with hTRPV4 colored in grey and RhoA colored in cyan. B) The overall structure of hTRPV4$_{A2}$-RhoA is viewed parallel to the membrane with one subunit shown in surface mode and the rest subunits in cartoon mode, with the same color scheme as in A. C) hTRPV4$_{A2}$-RhoA interface. D) Open-book representation of the interaction surfaces colored by Electrostatic potential with positive in blue and negative in red. E-F) Key molecular interactions between hTRPV4 and RhoA, with critical residues side chains involved in hTRPV4$_{A2}$-RhoA shown in the sticks with the corresponding density shown with blue mesh.

Table S1. Primer sequences

Target genes	Forward	Reverse
hTRPV4	AATTCAAAGGCCTACGTCGACatggcgga ttccagcgaaggcccc	CAGAACTTCCAGTGCGGCCGCgagc ggggcgtcatcagtcctcca
mTRPV4	CCCGGTCCGAAGCGCGCGGAATTcatgg cagatcctggtgatggtccc	GAACAGAACTTCCAGTGCGGCCgcc agtggggcatcgtccgtcctcca
hTRPV4-V469A	AAGTGGCGCAAGTTCGGGGCCGCCTC CTTCTACATCAACGTGGTC	GACCACGTTGATGTAGAAGGAGG CGGCCCCGAACTTGCGCCACTT
hTRPV4-S470A	TGGCGCAAGTTCGGGGCCGTCGCCTT CTACATCAACGTGGTCTCC	GGAGACCACGTTGATGTAGAAGG CGACGGCCCCGAACTTGCGCCA
hTRPV4-Y553A	GGCTCCTTCCAGCTGCTCgccTTCATCT ACTCTGTCCTG	CAGGACAGAGTAGATGAAggcGAG CAGCTGGAAGGAGCC
hTRPV4-Y591A	GGCTGGATGAATGCCCTTgccTTCACC CGTGGGCTGAAG	CTTCAGCCCACGGGTGAAggcAAG GGCATTCATCCAGCC
hTRPV4-F592A	TGGATGAATGCCCTTTACgccACCCGT GGGCTGAAGCTG	CAGCTTCAGCCCACGGGTggcGTAA AGGGCATTCATCCA
hTRPA4-D743A	TGGGCCACCACCATCCTGgccATTGAG CGCTCCTTCCCC	GGGGAAGGAGCGCTCAATggcCAG GATGGTGGTGGCCCA
hTRPV4-I744A	TGGGCCACCACCATCCTGGACgcTGAG CGCTCCTTCCCCGTATTC	GAATACGGGGAAGGAGCGCTCAgc GTCCAGGATGGTGGTGGCCCA

Table S2. Cryo-EM data collection, refinement, and validation statistics

	hTRPV4$_{GSK279}$ (EMD-36660) (PDB 8JU6)	hTRPV4$_{A1}$ (EMD-36659) (PDB 8JU5)	hTRPV4$_{A2}$ (EMD-36675) (PDB 8JVI)	hTRPV4$_{A2}$-RhoA (EMD-36676) (PDB 8JVJ)
**Data collection and processing**				
Magnification	130,000	130,000	130,000	
Voltage (kV)	300	300	300	
Electron exposure (e–/Å2)	60	60	60	
Defocus range (μm)	-1.0 ~ -2.0	-1.0 ~ -2.0	-1.0 ~ -2.0	
Pixel size (Å)	1.04	1.04	1.04	
Initial particle images (no.)	1,261,426	2,163,226	2,088,156	
Final particle images (no.)	206,679	102,087	48,492	47,803
Symmetry imposed	C4	C2	C2	C4
Map resolution (Å)	3.45	3.74	3.21	3.44
FSC threshold	0.143	0.143	0.143	0.143
Map resolution range (Å)	3.0-4.6	3.2-5.2	3.0-5.0	3.2-5.2
Refinement				
Initial model used (PDB code)	7XJ0	This study	This study	This study
Model resolution (Å)	3.6	3.9	3.5	3.9
FSC threshold	0.5	0.5	0.5	0.5
Map sharpening B factor (Å^2)	149	127	115	135
Model composition				
Non-hydrogen atoms	19,732	19,567	19,589	25,935
Protein residues	2464	2,435	2,414	3,216
Ligands	4	2	4	4
B factors (Å^2)				
Protein	79.46	101.06	49.91	111.83
Ligand	69.69	66.5	32.37	74.63
R.m.s. deviations				
Bond lengths (Å)	0.004	0.012	0.014	0.003
Bond angles (°)	0.668	0.663	0.706	0.625
Validation				
MolProbity score	1.74	2.01	2.02	1.74
Clashscore	10	12	13	10
Poor rotamers (%)	0.09	0	0	0.04
Ramachandran plot				
Favored (%)	96.65	92.88	92.5	96.2
Allowed (%)	3.35	7.04	7.45	3.8
Disallowed (%)	0	0.08	0.05	0

Note 1. Analytical information for A2**.**

ESI-MS m/z =573.05 [M+1]+; Calculated MW: 572.62

1H NMR (400 MHz, DMSO-d6) δ 11.68 (s, 1H), 8.71 (s, 2H), 8.53 (d, J = 2.3 Hz, 1H), 7.91 (dd, J = 8.6, 2.3 Hz, 1H), 7.17 (d, J = 8.5 Hz, 1H), 7.13 (s, 1H), 4.60-4.50 (br, 4H), 3.29 (d, J = 12.8 Hz, 2H), 2.60 (s, 3H), 2.54 (s, 3H), 1.98 – 1.86 (m, 2H), 1.62 (d, J = 7.7 Hz, 2H).

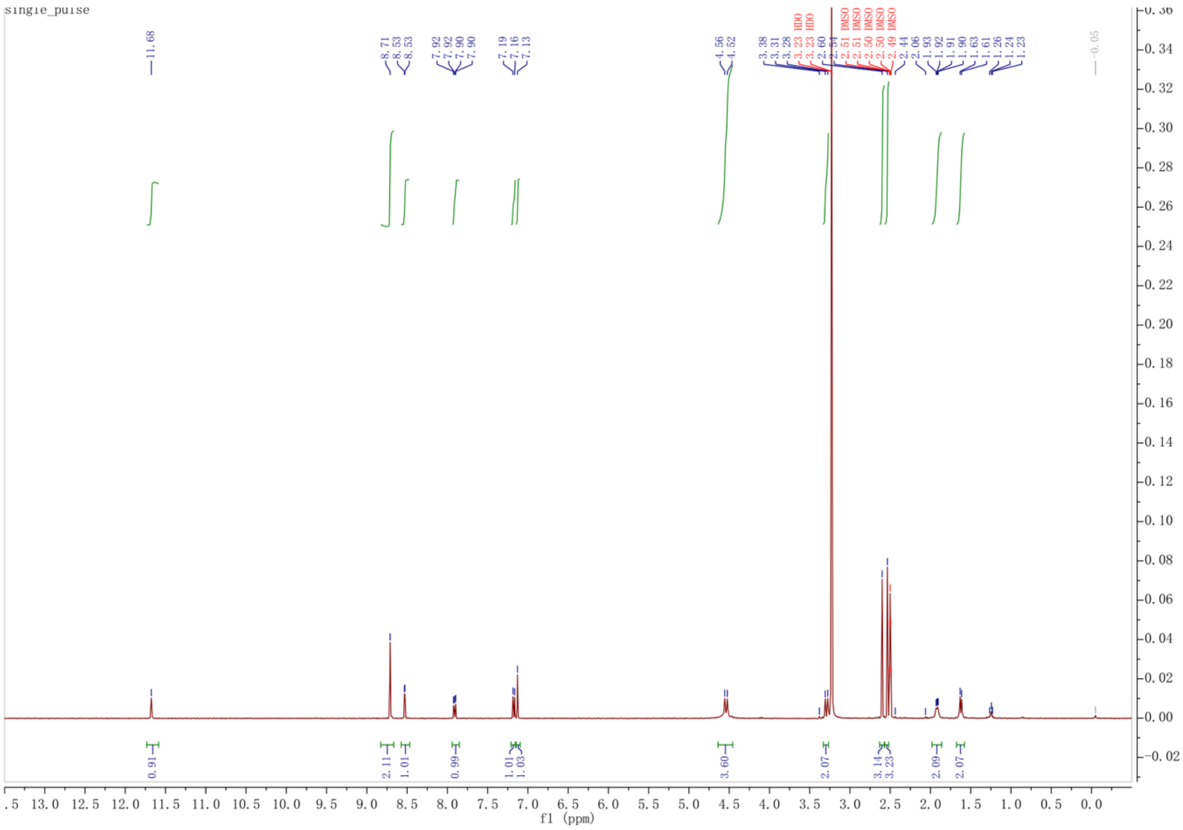

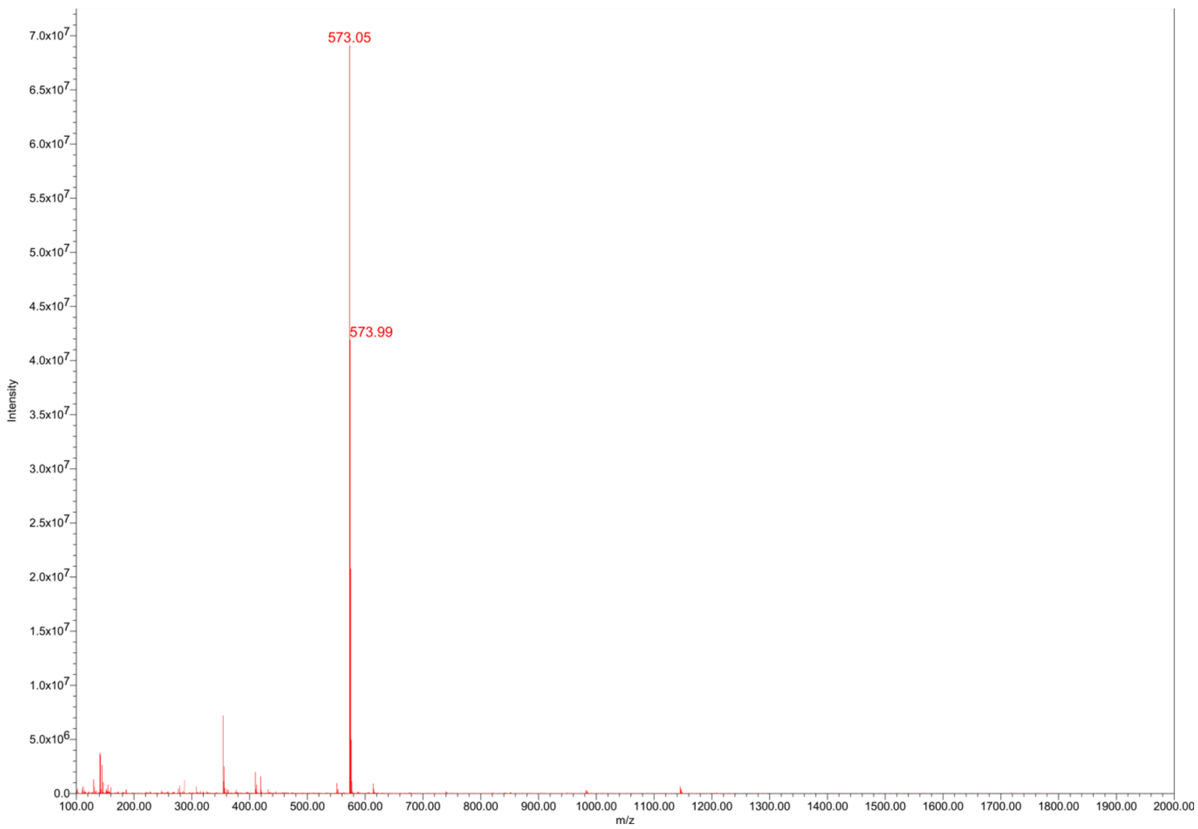
573.05
573.99
Intensity
m/z
7.0x10^7
6.5x10^7
6.0x10^7
5.5x10^7
5.0x10^7
4.5x10^7
4.0x10^7
3.5x10^7
3.0x10^7
2.5x10^7
2.0x10^7
1.5x10^7
1.0x10^7
5.0x10^6
0.0
100.00
200.00
300.00
400.00
500.00
600.00
700.00
800.00
900.00
1000.00
1100.00
1200.00
1300.00
1400.00
1500.00
1600.00
1700.00
1800.00
1900.00
2000.00
